# Identification of key genes and metabolites in thyroid eye disease through integrated transcriptomic and metabolomic analysis

**DOI:** 10.3389/fendo.2026.1844082

**Published:** 2026-09-07

**Authors:** Chengyan Fang, Qian Li, Nishan Zhao, Zhulin Hu, Chao Ma, Duozhang Xia, Xuesi Fan, Ping Xiang, LiWei Zhang, Hai Liu, Ji Yang

**Affiliations:** 1 Department of Ophthalmology, the Eye Disease Clinical Medical Research Center of Yunnan Province, the Eye Disease Clinical Medical Center of Yunnan Province, the Affiliated Hospital of Yunnan University, Kunming, China; 2 Department of Pathology, Affiliated Hospital of Yunnan University, Kunming, China; 3 Institute of Environmental Remediation and Human Health, School of Ecology and Environment, Southwest Forestry University, Kunming, China

**Keywords:** biomarker, machine learning, metabolomic, thyroid eye disease, transcriptomic

## Abstract

**Introduction:**

Thyroid eye disease (TED) markedly compromises ocular function and quality of life, imposing significant healthcare and economic burdens. Present therapeutic strategies, which mainly include glucocorticoids, teprotumumab, immunosuppressive drugs, and surgical treatments, exhibit limited effectiveness and are associated with high rates of relapse. Consequently, unraveling the molecular mechanisms of TED and discovering new diagnostic and therapeutic targets hold immense clinical importance.

**Methods:**

In this study, we performed an in-depth analysis of the transcriptomic and metabolomic landscapes of orbital adipose tissue in individuals with TED, aiming to fill the gap in comprehensive molecular characterizations and metabolic perturbations in this disease. By adopting a multi-omics integration strategy, we leveraged transcriptome sequencing, broad-spectrum metabolomics, weighted gene co-expression network analysis (WGCNA), machine learning, immune cell infiltration profiling, molecular docking, QPCR, and immunohistochemistry for biomarker identification. These techniques were systematically employed to pinpoint key genes and metabolites associated with TED, leading to the development of an accurate disease prediction model.

**Results:**

Remarkably, our analysis of the PRJNA1314138 dataset revealed 4, 250 differentially expressed genes (DEGs), with BPIFA1 and SERPINB3 identified as crucial genes through machine learning algorithms. These genes demonstrated outstanding predictive performance in both internal [Area under the curve (AUC)=0.953] and external dataset validations (AUC = 0.717). Furthermore, metabolomic profiling detected 2, 158 metabolites, pinpointing three key metabolites: gluconic acid, colneleic acid, and isoleucine-methionine. The integration of transcriptomic and metabolomic data underscored the significant enrichment of the tyrosine metabolism pathway, establishing functional links between the identified genes and metabolites.

**Conclusion:**

These insights offer novel perspectives on the molecular foundations of TED and propose that BPIFA1 and SERPINB3 could serve as promising biomarkers, while gluconic acid, colneleic acid and isoleucine-methionine may hold potential as metabolic indicators. Our research provides a solid framework for comprehending the pathophysiology of TED.

## Introduction

Thyroid Eye Disease (TED) is a complex autoimmune condition primarily associated with Graves’ disease, characterized by inflammation of the orbital tissues, leading to significant ocular complications such as proptosis and diplopia. The impact of TED extends beyond ocular symptoms, significantly diminishing the quality of life for affected individuals and imposing substantial healthcare costs on society (1, 2). The estimated incidence rate is 4.2 per 100, 000 person-years (3). Timely diagnosis and intervention play a pivotal role in enhancing prognosis and improving patients’ quality of life. In the absence of early detection and treatment, mild cases of TED have the potential to escalate into more severe forms of the condition (4). Current treatment modalities, including glucocorticoids, teprotumumab, immunosuppressive medications and surgical interventions, often yield unsatisfactory outcomes, with many patients experiencing persistent symptoms or recurrence of the disease (5, 6). This highlights an urgent need for a deeper understanding of the pathophysiological mechanisms underlying TED to identify novel therapeutic targets and improve clinical management strategies (7).

Previous research has primarily concentrated on the immune and inflammatory facets of TED. A substantial body of research has demonstrated that the thyroid-stimulating hormone receptor (TSH-R) and insulin-like growth factor-1 receptor (IGF-1R) are considered to play a pivotal role in TED (8, 9). Investigations into the pathogenesis of TED have predominantly centered on orbital fibroblasts (OFs), given their status as one of the primary target cells implicated in the disease’s pathological progression (10). Studies have demonstrated that when immune cells are activated and inflammatory cytokines are secreted, fibroblasts undergo differentiation into adipocytes and myofibroblasts. These differentiated cells synthesize copious amounts of extracellular matrix (ECM), ultimately leading to tissue remodeling and fibrosis characteristic of TED (11, 12). With the advancement of multi-omics technologies, whole-genome sequencing (WGS), DNA methylation assays, transcriptomics, metabolomics, and single-cell sequencing technologies have been employed to search for key biomarkers of TED (13). Based on peripheral blood samples and periorbital tissue sequencing, it has been found that LDLR, CDK5, and PIK3CB are correlated with the TED phenotype (14). Recent research has indicated that the dysregulation of metabolic pathways and alterations in gene expression patterns within orbital tissues could be of paramount importance in the onset and progression of TED (15). Metabolic profiling has unveiled a marked upregulation of glycolysis-related parameters in the orbital fibroblasts of TED patients (13). Nevertheless, when it comes to current TED research, a mere 33 studies have employed transcriptomics, while only 5 have utilized metabolomics and lipidomics (13). Furthermore, it’s worth noting that only a subset of these studies have utilized periorbital tissues or extraocular muscles. Notably, there have been no reports on studies that have combined transcriptomic and metabolomic analyses of periorbital tissues to elucidate the pathogenesis of TED. However, a significant knowledge gap still persists regarding the molecular transcriptional and metabolic changes that transpire within orbital tissues affected by this disease (16). Moreover, integrating transcriptomic and metabolomic methodologies could offer a holistic understanding of the disease mechanisms, thereby laying the groundwork for the discovery of biomarkers.

**Figure 1:**
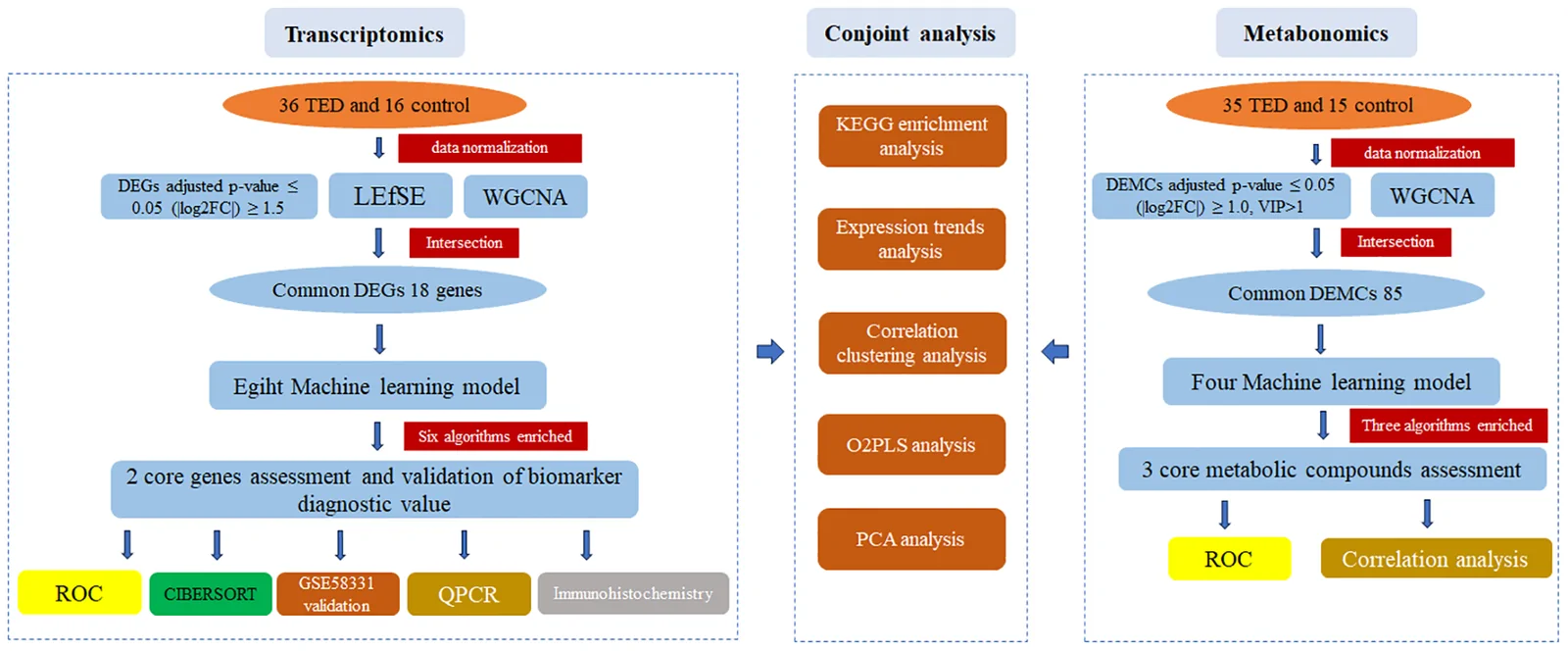
Research design flow chart.

This study employs a multi-omics approach, integrating transcriptome sequencing and metabolome analysis to explore the changes in gene expression and metabolic profiles within orbital fat tissues from TED patients. This methodology not only allows for the identification of differentially expressed genes and metabolites but also facilitates the construction of a predictive diagnostic model for TED. By focusing on the interactions between genetic and metabolic alterations, this research aims to elucidate the complex molecular networks involved in TED pathogenesis (17). Furthermore, the study utilizes advanced statistical methods, WGCNA and machine learning algorithms to analyze the multi-omics data, enhancing the precision of biomarker discovery and the development of personalized treatment strategies for TED patients (18, 19). At the same time, QPCR and immunohistochemistry techniques were also used to verify the key biomarkers at the tissue level. **Figure 1** depicts our research protocol.

The proposed research aims to fill the existing knowledge gap regarding the molecular mechanisms of TED by employing a comprehensive multi-omics strategy. This innovative approach has the potential to unveil novel insights into the disease and contribute to the development of more effective diagnostic and therapeutic strategies for patients suffering from TED (20, 21). Our article is compliant with the TITAN Guidelines 2025 - governing declaration and use of AI (22).

## Methods

### Datasets acquisition and patient demographics

Currently, for patients with orbital fat prolapse that affects their appearance in clinical practice, we need to perform orbital fat prolapse resection to improve their appearance. For patients with TED who have apical compression and significant proptosis along with incomplete eyelid closure, we need to carry out orbital decompression surgery. This procedure aims to enlarge the bony orbital space while removing the hyperplastic orbital fat tissue both inside and outside the muscular cone to relieve orbital pressure and save the patient’s vision. The tissues removed during surgery are typically considered waste tissues. However, with the advancement of omics technologies, such tissues can be sequenced to explore the patterns of disease occurrence and development, providing new insights into disease pathogenesis and laying the foundation for targeted therapies.

The study protocol received approval from the Ethics Review Committee, and the research was conducted in strict accordance with the Declaration of Helsinki. In this study, orbital adipose tissue specimens were collected through a non-randomized approach, without implementing blinding procedures. The inclusion criteria for the study participants were as follows: (1) Patients free from other inflammatory conditions; (2) Patients who had not undergone thyroidectomy; (3) Patients exhibiting normal thyroid function following anti-thyroid therapy; (4) Patients with pronounced symptoms of eye irritation or ocular motility disorders that necessitated surgical intervention. (5) The disease in patients with TED is in a relatively quiescent phase, with a GAS (clinical activity score) of less than three. (6) All the patients selected for TED and orbital fat prolapse did not have a history of using teprotumumab or steroids. The baseline data of the patients are presented in **Supplementary Table 1**, including disease severity, disease duration, smoking status, thyroid function, thyroid autoantibody levels. The patients have a disease duration of more than one year and exhibit progressive visual deterioration, and CT (computerized tomography) scans reveal compression of the optic nerve. During the surgical procedure, orbital adipose tissue samples were procured for subsequent analysis. All surgical operations were carried out by a single surgeon under general anesthesia, with the operative time lasting 2–3 hours, and anesthetic agents such as propofol were administered during the procedure. All the patients undergoing surgery received combined intravenous and inhalation anesthesia, using drugs such as propofol, sevoflurane, sufentanil, and epinephrine. The study cohort consisted of 36 patients who underwent orbital decompression surgery, with a gender distribution of 17 males and 19 females. The mean age of the patients was 51.5 years, and the average duration of their illness was 1.91 years. The control group comprised patients diagnosed with simple orbital fat prolapse, with a total of 16 cases included. All patients in the control group underwent orbital fat prolapse surgery. The inclusion criteria for the control group were: (1) No history of TED or other thyroid related disorders; (2) Voluntary provision of the excised fat tissue. The control group consisted of 7 females and 9 males, with an average age of 55.1 years and an average disease duration of 1.5 years, the detailed patient information can be found in **Supplementary Table 1**.

Our RNA-seq data have been deposited in Sequence Read Archive (SRA) with accession numbers PRJNA1314138. Previously published public dataset used for external validation can be accessed through NCBI’s Gene Expression Omnibus (GEO) under the accession number GSE58331 (available at: https://www.ncbi.nlm.nih.gov/geo/). For validation purposes, we selected 27 anterior orbital tissue samples from patients diagnosed with TED and 22 samples of normal anterior orbital tissue from the GSE58331 dataset (23, 24).

### Transcriptome sequencing

Orbital adipose tissue samples were obtained from 16 individuals in the control group and 36 patients diagnosed with TED. These samples were subsequently stored in the biospecimen repository at a temperature of -196 °C (immersed in liquid nitrogen). For both control and TED samples, total RNA was extracted from the orbital adipose tissue utilizing the Tissue RNA Extraction Kit 2.0 Plus (Vazyme, Nanjing, China). For RNA quality inspection, we utilize the Qubit 4.0 Fluorometer (Thermo Fisher Scientific, Massachusetts, USA, Model: Qubit4.0) and the Qsep400 High-throughput Nucleic Acid and Protein Analysis System (BioOptic, Taiwan, China, Model: QSEP400), with the RNA Integrity Number (RIN) determined to be 5.6. For each bulk RNA-Seq preparation, 240 ng of RNA was employed as the starting material. Hieff NGS^®^ Ultima Dual-mode mRNA Library Prep Kit for Illumina^®^ was used in the preparation process (25).

Sequencing was conducted on the Illumina NovaSeq 6000 platform, with a read length of 150 base pairs (bp). We use fastp to filter the data, employ Hisat2 for reference genome alignment, and utilize featureCounts for quantification to obtain the gene expression matrix. After the company processed the fastp format files, the gene expression matrix was generated for subsequent analysis (26).

### Broad target metabolomics

In this study, orbital tissues from paired with the transcriptome 35 TED patients and 15 control subjects underwent comprehensive metabolic profiling. Broad-target metabolomics analysis was performed using liquid chromatography-tandem mass spectrometry (LC–MS). Samples stored at -196 °C were thawed on ice, homogenized with a grinder (30 Hz, 20 s), and mixed with 400 μL of methanol-water solution (7:3, V/V) containing an internal standard. The mixture was shaken (2500 rpm, 5 min), cooled on ice (15 min), and centrifuged (12000 rpm, 10 min, 4 °C). A 300 μL supernatant was collected, incubated at -20 °C for 30 min, and centrifuged again (12000 rpm, 3 min, 4 °C). Finally, 200 μL aliquots of supernatant were prepared for LC-MS analysis. To maximize metabolite coverage, data were acquired separately in positive and negative ion modes using a high-resolution QTRAP^®^6500+ mass spectrometer (SCIEX, China). LC–MS data were processed with Analyst 1.6.3 software. The system, equipped with an ESI Turbo Ion-Spray interface, operated in both ion modes and was controlled by Analyst 1.6.3. Key ESI parameters included a source temperature of 500 °C, ion spray voltages of 5500 V (positive) and -4500 V (negative), and gas settings of 55 psi (GSI), 60 psi (GSII), and 25.0 psi (CUR), with high collision gas (CAD). Instrument tuning and mass calibration were performed using 10 and 100 μmol/L polypropylene glycol solutions in QQQ and LIT modes, respectively. Specific multiple reaction monitoring (MRM) transitions were monitored for each elution period (27, 28). Data preprocessing, statistical analysis, and metabolite taxonomic and functional annotation were conducted using the metabolomics R package metaX (29), Metware online analysis software (https://cloud.metware.cn/#/home), and the metabolome information analysis process. Quality control (QC) samples, created by pooling equal volumes of supernatant from all samples, were used to evaluate the stability of experimental results. All metabolite annotations were generated in conjunction with the database of Metware.

### Identification of differentially expressed genes and differentially expressed metabolic compounds

In this analysis, orbital tissues from patients with TED were designated as the experimental group, whereas orbital tissues derived from individuals with orbital fat prolapse formed the control group. For the rigorous determination of DEGs, we implemented a Benjamini–Hochberg False Discovery Rate (FDR) multiple testing correction. To gauge the extent of differences between the two sample groups, we conducted principal component analysis (PCA). To identify DEGs within the PRJNA1314138 dataset, we utilized the R programming. Genes were deemed differentially expressed if they met the following criteria: an adjusted p-value ≤ 0.05 and an absolute log2 fold change (|log2FC|) ≥ 1.5. Additionally, volcano plots were generated to visually depict the DEGs, with the same thresholds applied for highlighting significant genes. The dataset underwent normalization to ensure consistency across samples.

The metabolomics data were subjected to upstream analysis to obtain the expression matrix. Then, the visualization of PCA, orthogonal two-way partial least squares (O2PLS), and volcano plots, was conducted using the MetWare online analysis platform (accessible at: https://cloud.metware.cn/#/home). Metabolomic data were analyzed with R version 4.2.1, employing packages such as ComplexHeatmap, MetaboAnalystR, corrplot and ggraph. To visualize and pinpoint key metabolic changes, PCA was applied to reduce the dimensionality of the original multivariate dataset, comprising 15 control samples and 35 samples from TED patients, enabling an initial comparison of global metabolic differences between the two groups. Subsequently, a more refined screening of differential metabolites was achieved by integrating multiple analytical methods. Variable importance in projection (VIP) value was extracted from orthogonal partial least squares discriminant analysis (OPLS-DA), with score and permutation plots generated using the MetaboAnalystR package. Metabolites were designated as DEMCs if they met the following criteria: a VIP score greater than 1, an absolute log2-transformed fold change (|log2FC|) of at least 1, and an adjusted P-value <0.05.

### GO and KEGG pathway enrichment analysis in transcriptomics and metabolomics

To elucidate the functions of DEGs and DEMCs, a functional enrichment analysis was performed. Initially, gene ontology (GO) analysis was employed to explore gene functions across three domains: cellular component (CC), molecular function (MF), and biological process (BP). Subsequently, the Kyoto Encyclopedia of Genes and Genomes (KEGG) pathway analysis was utilized to gain insights into the biological pathways associated with these genes (30, 31). Furthermore, the R language (version 4.2.1) package “clusterProfiler” was employed to execute the enrichment analysis and visualize the results (32). The criteria for statistical significance were set at a p-value threshold of less than 0.05, and a minimum enrichment gene count of 2 was required for a pathway or term to be considered significantly enriched. Finally, the MetWare online analysis platform (accessible via: https://cloud.metware.cn/#/home) was employed to conduct KEGG pathway enrichment analysis within the metabolomics dataset, as well as to perform an integrative analysis combining transcriptomic and metabolomic data. Metware was designed based on R 4.2.1 program. This approach facilitated a comprehensive exploration of the functional pathways and molecular interactions underlying the biological phenomena under investigation.

### Modular genes and metabolic compounds identification using WGCNA

The Weighted Gene Co-expression Network Analysis (WGCNA) package was utilized to perform weighted correlation network analysis (33). Within the WGCNA framework, an optimal power value was determined to construct both a weighted adjacency matrix and a topological overlap matrix (TOM). This process facilitated the capture of complex gene-gene and metabolite-metabolite relationships. To focus on the most informative components of the dataset, WGCNA analyses were restricted to the top 10% of genes and the top 25% of metabolic compounds exhibiting the highest variance across samples. The WGCNA methodology was further enriched by integrating clinical trait-module correlations, enabling the identification of expression patterns closely associated with specific phenotypic outcomes. For network construction and module detection, a comprehensive dataset comprising 23, 881 genes and 2, 158 metabolic compounds was employed.

### Linear discriminant analysis effect size (LEfSE) algorithm

R program 4.3.3 and R package “reshape2” and “microeco” are used to LEfSe analysis. LEfSE is a bioinformatics-based tool designed to identify differences in feature abundance among samples from different groups. Initially, the Kruskal-Wallis rank-sum test is employed to compare the abundance of gene features across groups, thereby screening out features with significant differences (where p < 0.01 indicates statistical significance) (34, 35). Subsequently, linear discriminant analysis (LDA) is applied to quantify the magnitude of these differences and calculate the LDA scores (LD scores). Finally, the top 50 features with the highest LDA scores are selected as candidate markers, focusing on the top 50 gene features that exhibit the most significant differences between groups (36). Using LEfSE, we analyzed the differential gene data between the TED group and the control group, and the top 50 features identified were utilized for subsequent screening of hub genes.

### Machine learning model screens the candidate genes and metabolic compounds

Pivotal genes involved in the interactions among DEGs, WGCNA modular genes, and the top 50 LEfSE genes were further selected and evaluated via eight machine learning algorithms: bagged trees, bayesian methods, random forest, boruta, learning vector quantization (LVQ), support vector machine (SVM), XGBoost, and a least absolute shrinkage and selection operator (LASSO) approach with repeated 10-fold cross-validation with 1000 repetitions (37, 38). DEGs identified as feature genes by at least six algorithms, based on classification performance, were deemed significant hub genes within the interactions among DEGs, WGCNA modular genes, and the top 50 LEfSE genes. The interactions among these key intersecting genes were subsequently visualized using the R package “UpSetR”. Based on the hub genes, was developed to predict the prevalence of TED. Receiver operating characteristic (ROC) curves were generated using the R package “pROC” to evaluate the diagnostic accuracy of the model for TED onset. Comprehensive validation techniques were applied to confirm the model’s performance in distinguishing TED from control samples. Additionally, the GSE58331 dataset was incorporated for external validation of the model’s predictive capabilities.

For metabolomics data, determine the intersection between module metabolites identified through WGCNA screening and DEMCs. Subsequently, apply machine learning screening to the resulting differential metabolites. Four machine learning models were developed using the “caret” R package (39), including the Generalized Linear Model (GLM), Random Forest (RF), eXtreme Gradient Boosting (XGB), and Support Vector Machine (SVM). These base learners were trained on the subsets, and their predictive outputs were combined to generate the final output of the integrated model [14]. The dataset, comprising 15 control samples and 35 TED samples, was randomly partitioned into a validation set (30%, n = 15) and a training set (70%, n = 35). The “DALEX” package was employed to analyze the four machine learning models, generating visualizations of residual distribution and feature importance. The “caret” R package automatically optimized the model parameters, which were assessed using repeated 10-fold cross-validation with 1000 repetitions with default parameters. The “pROC” package was utilized to visualize the area under the ROC curves. For DEMCs, the intersection of the top 10 DEMCs from each algorithm was calculated, and metabolites present in the intersections of any two algorithms were designated as key metabolites.

### Exploring immune cell infiltration and hub gene-immune cell links

Leveraging the CIBERSORT deconvolution algorithm (40), we systematically evaluated the infiltration patterns of 22 distinct immune cell types in both the TED and control groups, and subsequently compared these patterns to identify any significant differences. Specifically, we employed the CIBERSORT method to quantitatively assess gene expression levels, thereby determining the extent of immune cell infiltration among patients. The immune cell populations analyzed included both immune-stimulatory and immune-suppressive cells. To investigate the interrelationships among these immune cells, we performed a correlation analysis using the Spearman coefficient and visualized the results through correlation heatmaps. Additionally, we explored the associations between hub genes and immune cells. Statistical significance was defined as p < 0.05.

### QPCR analysis of BPIFA1 and SERPINB3 expression

The method is detailed in the previous literature (41), orbital fat tissue specimens were collected as validation samples, comprising 8 pairs from orbital fat prolapse and 8 pairs from patients diagnosed with TED. Following homogenization in liquid nitrogen using TRIzol reagent (Thermo Fisher Scientific), total RNA was isolated with a commercial extraction kit (Vazyme). Complementary DNA (cDNA) synthesis was subsequently performed using a reverse transcription kit (TaKaRa Bio Inc.). Relative expression levels of target genes (BPIFA1 and SERPINB3) were quantified by RT-qPCR using TB Green Fast qPCR Mix (TaKaRa Bio Inc.), normalized against the reference gene GAPDH via the 2^(−ΔΔCT) calculation method. Specific primer sequences are detailed in **Supplementary Table 2**.

### Immunohistochemical analysis of BPIFA1 and SERPINB3

Experiment according to our previous method (41), orbital fat tissues from patients with TED and orbital fat prolapse were processed for immunohistochemical analysis of BPIFA1 and SERPINB3 using primary antibodies from HUABIO (China). Following deparaffinization, rehydration, and antigen retrieval, sections were blocked for endogenous peroxidase activity and non-specific protein binding, then incubated with primary antibodies overnight at 4 °C. After rinsing, a biotinylated secondary antibody or polymer-based system was applied, followed by visualization with DAB chromogen and counterstaining with hematoxylin. The stained sections were examined under a light microscope to compare the expression patterns of BPIFA1 and SERPINB3 between the two groups, providing insights into their potential roles in TED and orbital fat prolapse pathogenesis.

### Identification of interacting drugs

To pinpoint promising drug capable of modulating the expression or function of specific genes, we utilized the DSigDB database via the Enrichr platform. This comprehensive database aggregates drug signatures from diverse sources, enabling us to pinpoint compounds reported to influence the expression or activity of our target genes. We gathered detailed information on the active compounds, including their names, molecular weights, and 2D structural formulas. The 3D protein structures were sourced from the PubChem (URL: https://pubchem.ncbi.nlm.nih.gov/) and UniProt (URL: https://www.uniprot.org/) repositories. Subsequently, we prepared both ligands and proteins for molecular docking using the AutoDock online tool (URL: https://cadd.labshare.cn/cb-dock2/php/blinddock.php). The crystal structures of the target proteins underwent refinement, which involved removing water molecules, adding hydrogen atoms, modifying amino acids, optimizing energy configurations, and adjusting force field parameters. Following this, we performed virtual docking screenings using Vina within the PyRx software suite. The Binding Affinity values (expressed in kcal/mol), which quantify the interaction strength between ligands and receptors, were used to assess binding stability, with lower values indicating more robust binding. For additional analysis, we employed Discovery Studio, while PYMOL was used for visualizing the docking results.

### MOFA multi-omics joint analysis

A systematic analysis of transcriptomic and metabolomic data from patients with TED and orbital fat prolapse was conducted using the MOFA (Multi-Omics Factor Analysis) integrative approach. A total of 2, 158 metabolites were included; transcriptomic data were derived from the control group and the TED group. For analysis, the top 5, 000 highly variable genes were filtered to ensure computational efficiency. All analyses were performed using R software (version 4.3.3) with the. Data preprocessing employed Log2 transformation combined with Z-score normalization to stabilize variance and eliminate scale differences across omics layers. Principal component decomposition was carried out using the prcomp function, and the first 10 principal components were extracted as latent factors. Factor loadings of key genes (BPIFA1, SERPINB3) and key metabolites (Gluconic acid, Colneleic acid, L-Isoleucine, L-Methionine, L-Tyrosine) were analyzed. Coordinated gene-metabolite signature between genes and metabolites were evaluated using Pearson correlation coefficients, with statistical significance assessed by two-sided tests and denoted as *p<0.05, **p<0.01, and ***p<0.001. Additionally, tyrosine-metabolism-related metabolites were identified to evaluate the regulatory effects of BPIFA1, SERPINB3 on tyrosine metabolism.

### Statistical analysis

Data visualization tasks, encompassing enrichment analysis, PCA, LEfSe, machine learning, immune cell infiltration, WGCNA and the creation of group comparison charts, were carried out utilizing R software versions 4.2.1 and 4.3.3. The reported results are presented as the mean ± standard deviation (SD). The normality of the data was assessed using the Shapiro–Wilk test, and the homogeneity of variances was evaluated using Levene’s test before applying parametric tests (Student’s t-test). To determine statistically significant differences between two samples subjected to distinct treatments, the Student’s t-test was applied. A p-value below 0.05 was deemed to indicate statistical significance. In the visual displays of data, asterisks are used to highlight the level of statistical significance, with the following conventions: *: P < 0.05; **: P < 0.01; ***: P < 0.001.

## Results

### Data correction and visualization of differential gene results

**Figure 2:**
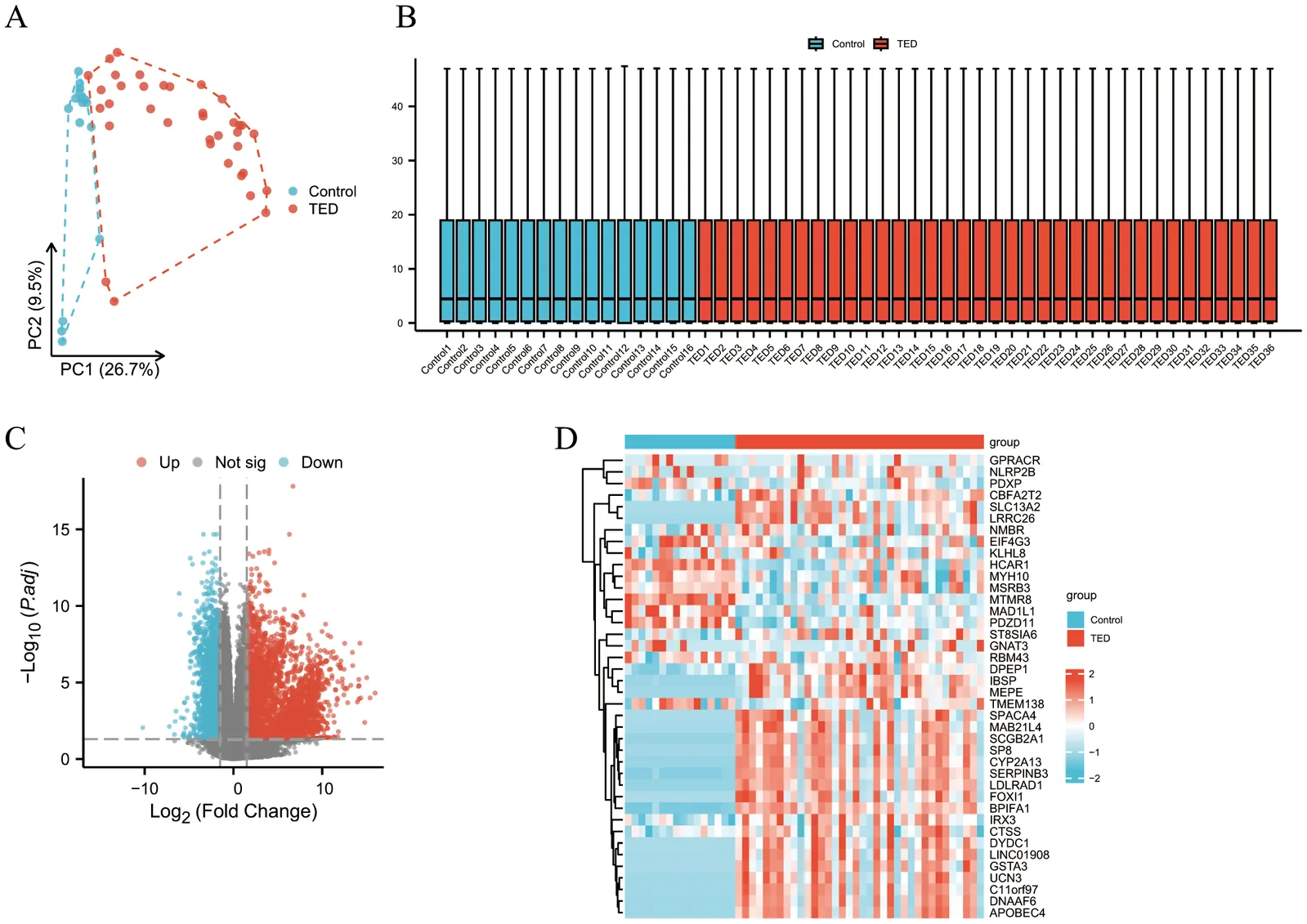
Profiling of differentially expressed genes (DEGs). **(A)** Principal component analysis (PCA) comparing control and TED groups. **(B)** Data normalization. **(C)** Visualization of DEGs using volcano plots. **(D)** Heat map representation of top 20 upregulated and downregulated genes.

PCA unveiled notable disparities in sample distribution patterns between the experimental and control groups (depicted in **Figure 2A**). In the PRJNA1314138 dataset, following normalization and a cross-comparable evaluation, it became apparent that the data distributions of both sample sets conformed to the established standard criteria. This conformity underscored the high quality and cross-comparability of the microarray data (as illustrated in **Figure 2B**). Leveraging the R programming tool and applying the criteria of an adjusted p - value ≤ 0.05 and an absolute log2 fold change (|log2FC|) ≥ 1.5, we successfully identified 4, 250 differentially expressed genes (DEGs) within the PRJNA1314138 dataset. Subsequently, we carried out cluster analysis on these identified DEGs. To present the key findings, we generated a heat map showcasing the top 20 upregulated and downregulated DEGs in the PRJNA1314138 dataset. Based on the cluster analysis of the dataset, we created both volcano plots and heat maps (displayed in **Figures 2C, D**). These visualizations not only highlighted the differentially expressed genes but also demonstrated a high level of confidence in the sample clustering results, reinforcing the reliability of our analytical approach.

### Enrichment analysis of GO terms and KEGG pathways

**Figure 3:**
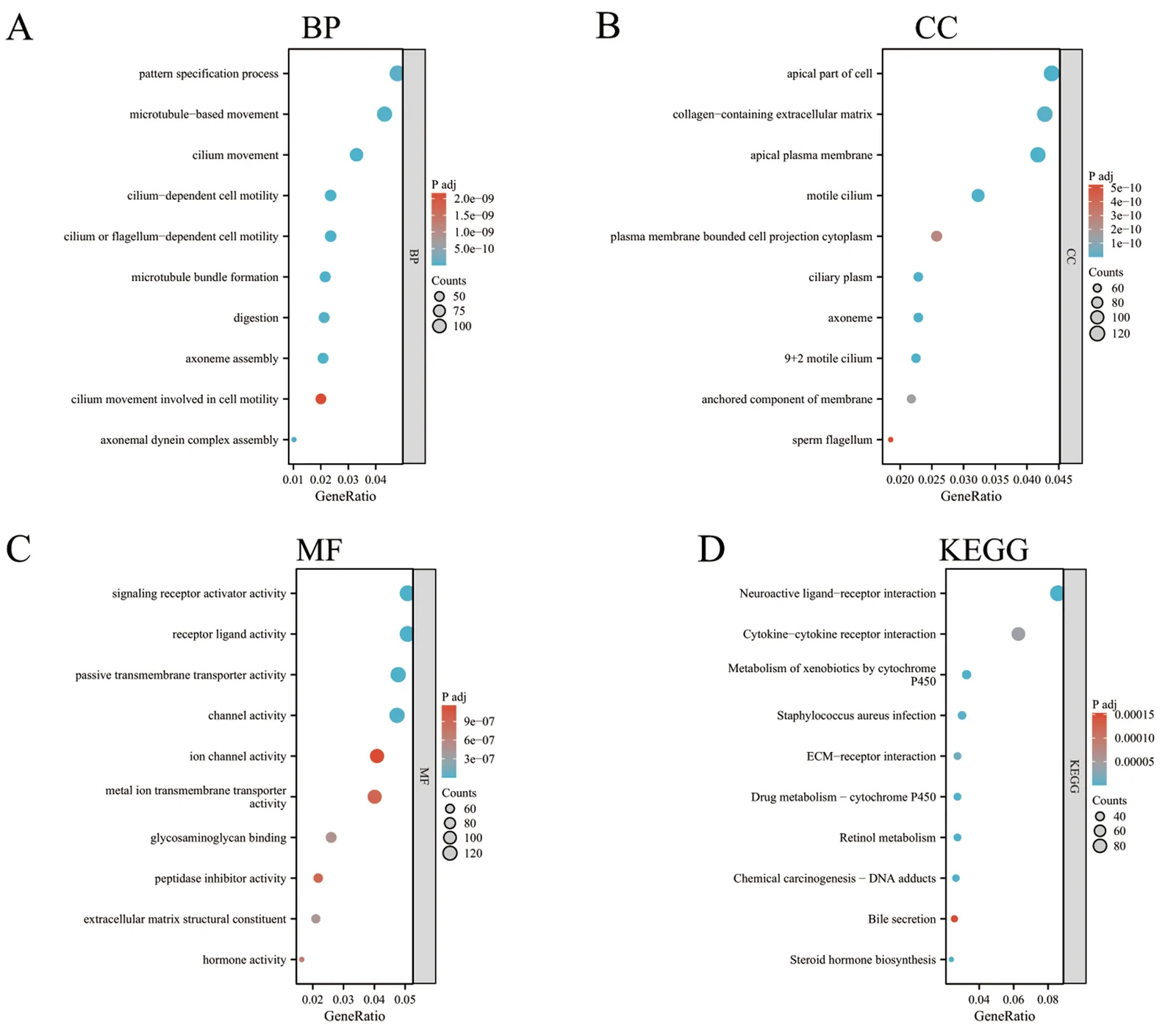
Enrichment analysis of GO terms and KEGG pathways. **(A–C)** Bubble graphs illustrating the top 10 enriched GO items, categorized into biological process (BP), cellular component (CC), and molecular function (MF). **(D)** Bubble diagram depicting the top 10 significantly enriched KEGG pathways.

A total of 4, 250 DEGs were identified in this study. Leveraging the GOKEGG database, we conducted a comprehensive enrichment analysis, which yielded 694 biological processes (BP), 92 cellular components (CC), and 149 molecular functions (MF). Furthermore, 30 KEGG pathways exhibited significant enrichment (p < 0.01, q < 0.05), providing insights into the underlying mechanisms characteristic of TED disease manifestation (**Figures 3A–C**).

The top 10 significantly enriched KEGG pathways comprised pathways involved in the metabolism of xenobiotics by cytochrome P450, neuroactive ligand-receptor interaction, retinol metabolism, drug metabolism by cytochrome P450, chemical carcinogenesis via DNA adducts, steroid hormone biosynthesis, ECM-receptor interaction, Staphylococcus aureus infection, cytokine-cytokine receptor interaction, and bile secretion. These pathways provide a broader context for understanding the systemic effects and pathological processes associated with TED (**Figure 3D**).

### WGCNA analysis of module gene enrichment results

**Figure 4:**
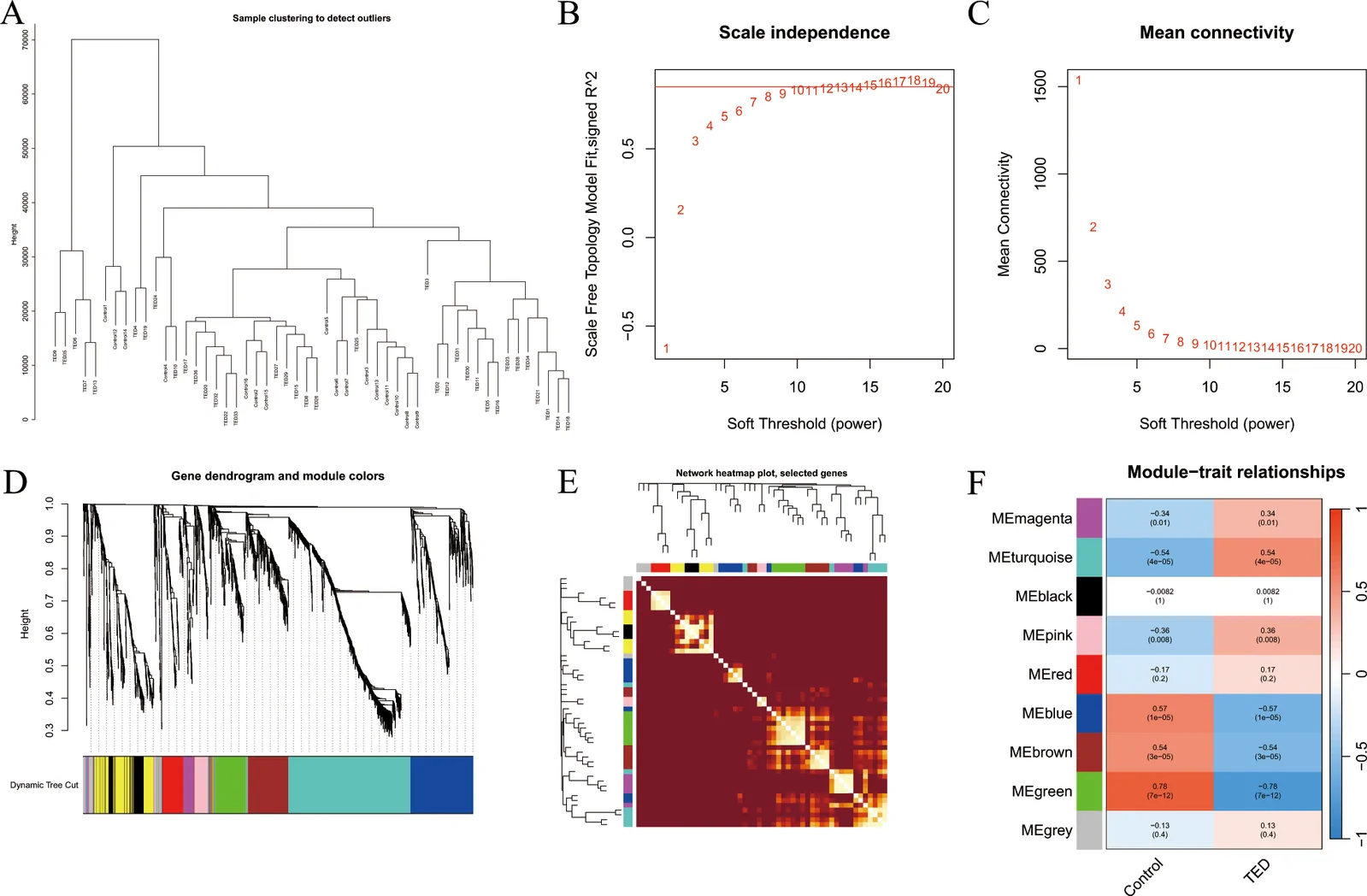
Modular genes based on co-expression patterns. **(A)** Dendrogram depicting the clustering of eigengenes. **(B, C)** Optimization of network parameters via the selection of an appropriate soft-thresholding power. **(D)** Gene dendrogram illustrating module identification through dynamic tree-cutting. **(E)** Heatmap displaying module assignments, with correlation patterns represented by distinct colors. **(F)** Matrix showing the correlation between eigengenes and traits, annotated with correlation coefficients and corresponding p-values.

Co-expression modules were constructed utilizing gene expression data derived from both TED and control samples, employing the WGCNA algorithm. For the ensuing analytical procedures, our focus was directed towards the top 10% of genes displaying the highest variability in expression levels. Cluster analysis of the samples was conducted using the “flashClust” package, a robust tool for hierarchical clustering. The power value was meticulously selected as 10, a choice that yielded an independence degree of 0.9 and a comparatively elevated mean connectivity degree (**Figures 4A–C**).

Leveraging the dynamic tree cutting algorithm, we successfully delineated ten distinct gene co-expression modules. Furthermore, we generated a heatmap representing the topological overlap matrix (TOM) for these modules (**Figures 4D, E**). Among these modules, the turquoise module emerged as particularly noteworthy due to its strongest correlation with TED. This module comprised 788 protein-coding genes (**Figure 4F**), suggesting its potential significance in the pathophysiology of TED.

### Establishment machine learning prediction of hub genes

**Figure 5:**
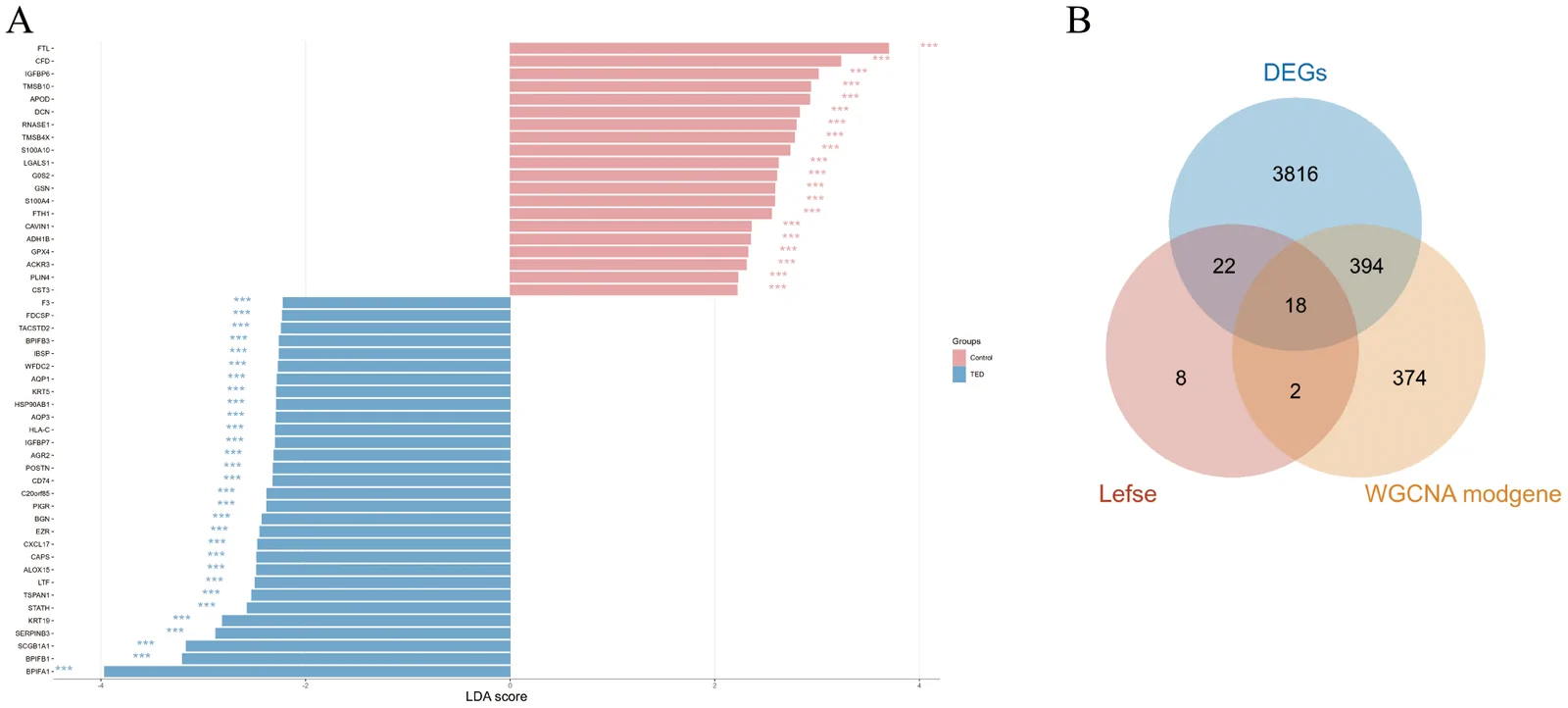
Determination of intersecting genes. **(A)** Utilizing the LEfSe algorithm, the top 50 genes distinguishing the TED group from the control group are identified. **(B)** Presentation of intersecting genes derived from the overlap among the LEfSe top 50 genes, WGCNA turquoise module genes, and DEGs.

**Figure 6:**
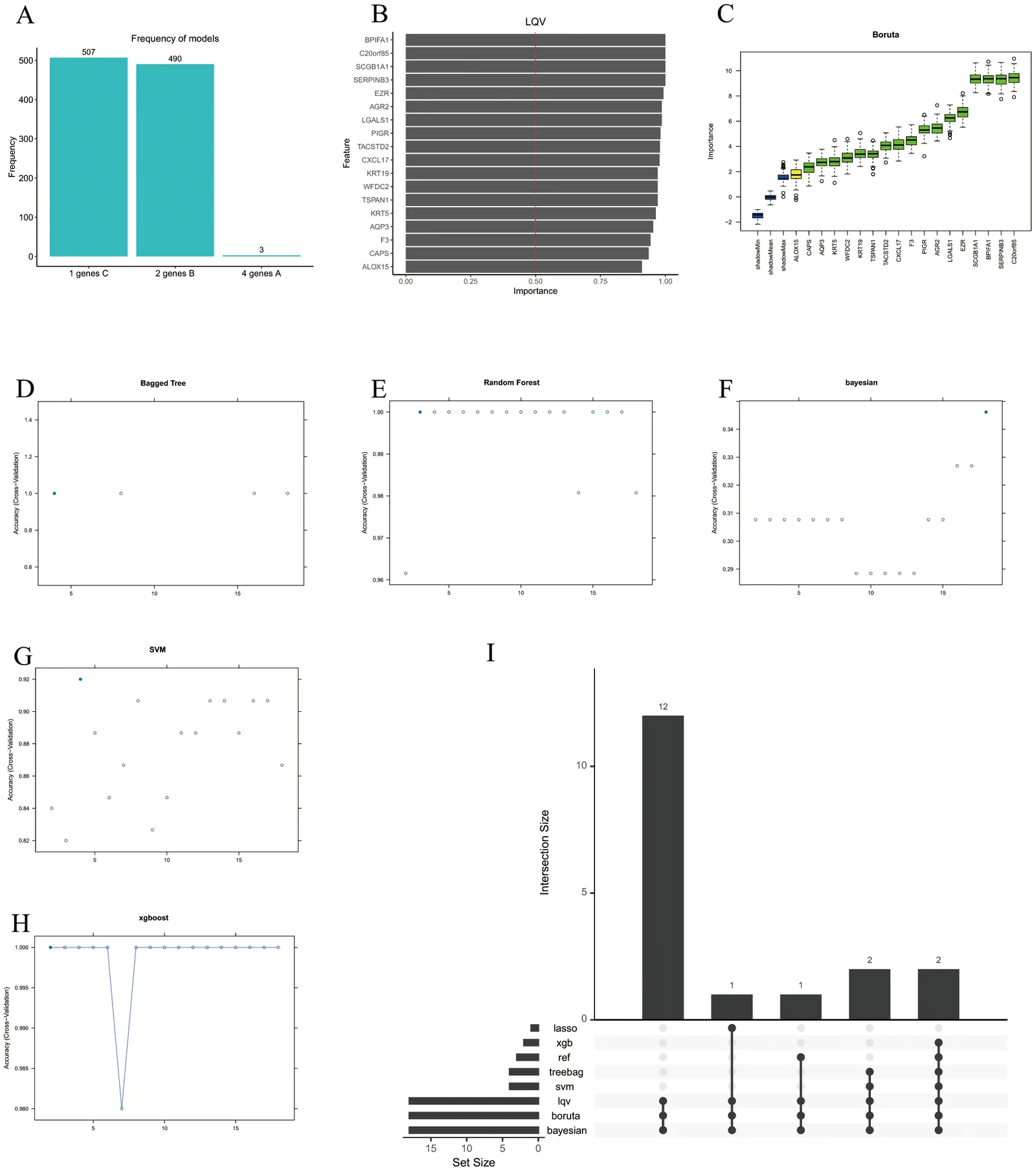
Screening of hub genes via machine learning. Results from various machine learning algorithms applied to the PRJNA1314138 dataset are displayed: **(A)** LASSO logistic regression model with repeated 10-fold cross-validation with 1000 repetitions, **(B)** Learning Vector Quantization (LVQ), **(C)** Boruta, **(D)** Bagged Trees, **(E)** Random Forest, **(F)** Bayesian Methods, **(G)** Support Vector Machine (SVM), **(H)** XGBoost. **(I)** A consolidated summary of genes recognized as significant for interactions by all eight machine learning algorithms.

**Figure 7:**
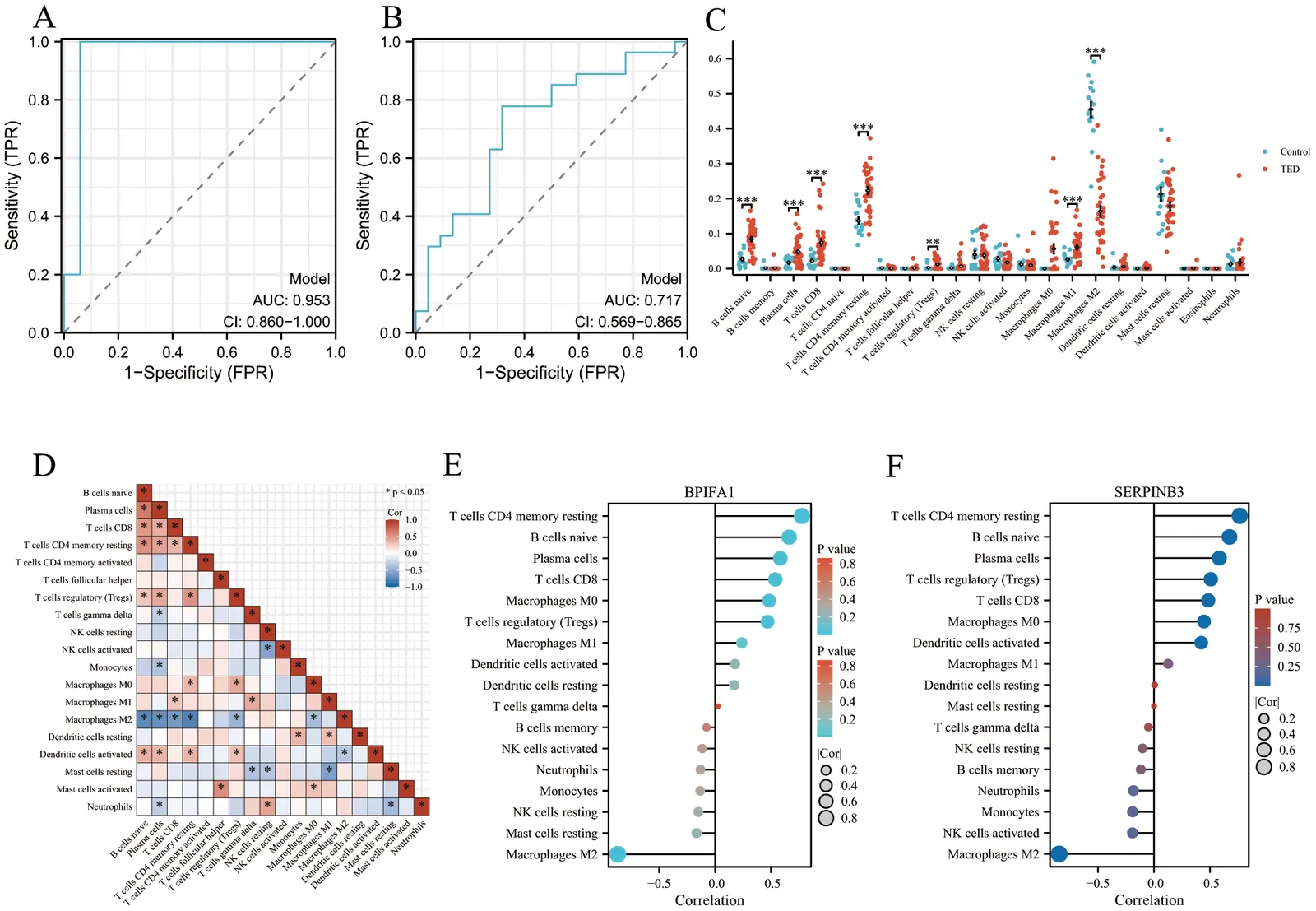
ROC curve evaluation and immune cell infiltration profiling. **(A, B)** Receiver operating characteristic (ROC) curve analysis was conducted to assess the diagnostic performance of a model incorporating the hub genes BPIFA1 and SERPINB3 across the PRJNA1314138 and GSE58331 datasets. **(C)** The relative abundance of 22 distinct immune cell types was quantified and compared TED and control samples. **(D)** A correlation matrix was generated to explore the interrelationships among 19 immune cell subsets. **(E, F)** The associations between the expression levels of BPIFA1 and SERPINB3 genes and the infiltration levels of various immune cells were examined and visualized. *: P < 0.05; **: P < 0.01; ***: P < 0.001.

By employing the LEfSe algorithm, we identified the top 50 genes exhibiting the most significant differences between the TED group and the control group (**Figure 5A**). The intersection of these 50 genes with the WGCNA turquoise module genes and DEGs yielded 18 genes of interest (**Figure 5B**). Machine learning algorithms were then trained using the PRJNA1314138 dataset to infer associations among these 18 genes, with model performance validated using the GSE58331 dataset. The results from various algorithms, including a LASSO logistic model with repeated 10-fold cross-validation with 1000 repetitions (**Figure 6A**), LVQ (**Figure 6B**), boruta (**Figure 6C**), bagged trees (**Figure 6D**), random forest (**Figure 6E**), bayesian methods (**Figure 6F**), SVM (**Figure 6G**), and XGBoost (**Figure 6H**), were intersected to identify genes closely linked to TED pathogenesis. This analysis highlighted 18 genes (**Figure 6I**): BPIFA1, SERPINB3, SCGB1A1, C20orf85, PIGR, KRT19, AGR2, KRT5, CXCL17, TSPAN1, ALOX15, WFDC2, TACSTD2, AQP3, CAPS, EZR, F3, and LGALS1, as being strongly associated with TED. Based on algorithm detection sensitivity and specificity analyses, the intersection genes jointly identified by the six algorithms exhibited high sensitivity. With a threshold of 5, two additional genes could be screened, increasing sensitivity by 11%, but at the cost of an increased false-positive risk (**Supplementary Figure 1**). Subsequently, genes consistently identified by more than six algorithms were considered as hub genes. Ultimately, BPIFA1 and SERPINB3 were selected as the key hub genes implicated in TED pathogenesis (**Figure 6I**). A diagnostic model was constructed based on the expression levels of the hub genes BPIFA1 and SERPINB3. Validation using the PRJNA1314138 and GSE58331 cohorts yielded an area under the curve (AUC) of 0.953 (95.3% confidence interval [CI]: 0.856–1.0) for the PRJNA1314138 cohort and an AUC of 0.717 (71.7% CI: 0.569–0.865) for the GSE58331 cohort, demonstrating robust generalizability across independent datasets (**Figures 7A, B**). To verify the stability of the diagnostic model, we calculated the Brier scores for the two datasets, with a Brier score of 0.154 for PRJNA1314138 and 0.2136 for GSE58331 (**Supplementary Figure 2**).

### CIBERSORT analysis of immune cell infiltration and hub gene correlations

The CIBERSORT deconvolution algorithm was utilized to precisely quantify the infiltration levels of 22 distinct immune cell subsets in both TED and control groups (**Figure 7C**). Notably, the immune infiltration analysis output contained numerous zero values for naïve CD4 T cells, eosinophils, and memory B cells, leading to their exclusion from subsequent immune cell correlation plots and hub gene immune cell correlation visualizations. Our analysis identified significant differences in the abundance of seven immune cell types between the two tissue groups. Specifically, naïve B cells, plasma cells, CD8 T cells, regulatory T cells, resting memory CD4 T cells, and M1 macrophages exhibited markedly higher levels in the TED group, whereas M2 macrophages were significantly more abundant in the control group (**Figure 7C**). Furthermore, a general correlation pattern was observed among the 19 immune cell types (**Figure 7D**).

Correlation analysis between these immune cells and BPIFA1 in TED revealed positive associations with naïve B cells (cor = 0.66, P = 8.24e-08), resting memory CD4 T cells (cor = 0.76, P = 1.48e-11), and plasma cells (cor = 0.58, P = 5.6e-6). Conversely, M2 macrophages demonstrated a significant negative correlation with BPIFA1 expression (cor = -0.87, P = 4.32e-17) (**Figure 7E**). Similarly, correlation analysis with SERPINB3 showed positive correlations with naïve B cells (cor = 0.68, P = 3.89e-08), resting memory CD4 T cells (cor = 0.77, P = 3.33e-11), regulatory T cells (cor = 0.51, P = 0.0001), and plasma cells (cor = 0.58, P = 5.5e-6), while M2 macrophages exhibited a significant negative correlation with SERPINB3 (cor = -0.85, P = 2.19e-15) (**Figure 7F**).

### Differentially expressed metabolites and metabolic pathway analysis

**Figure 8:**
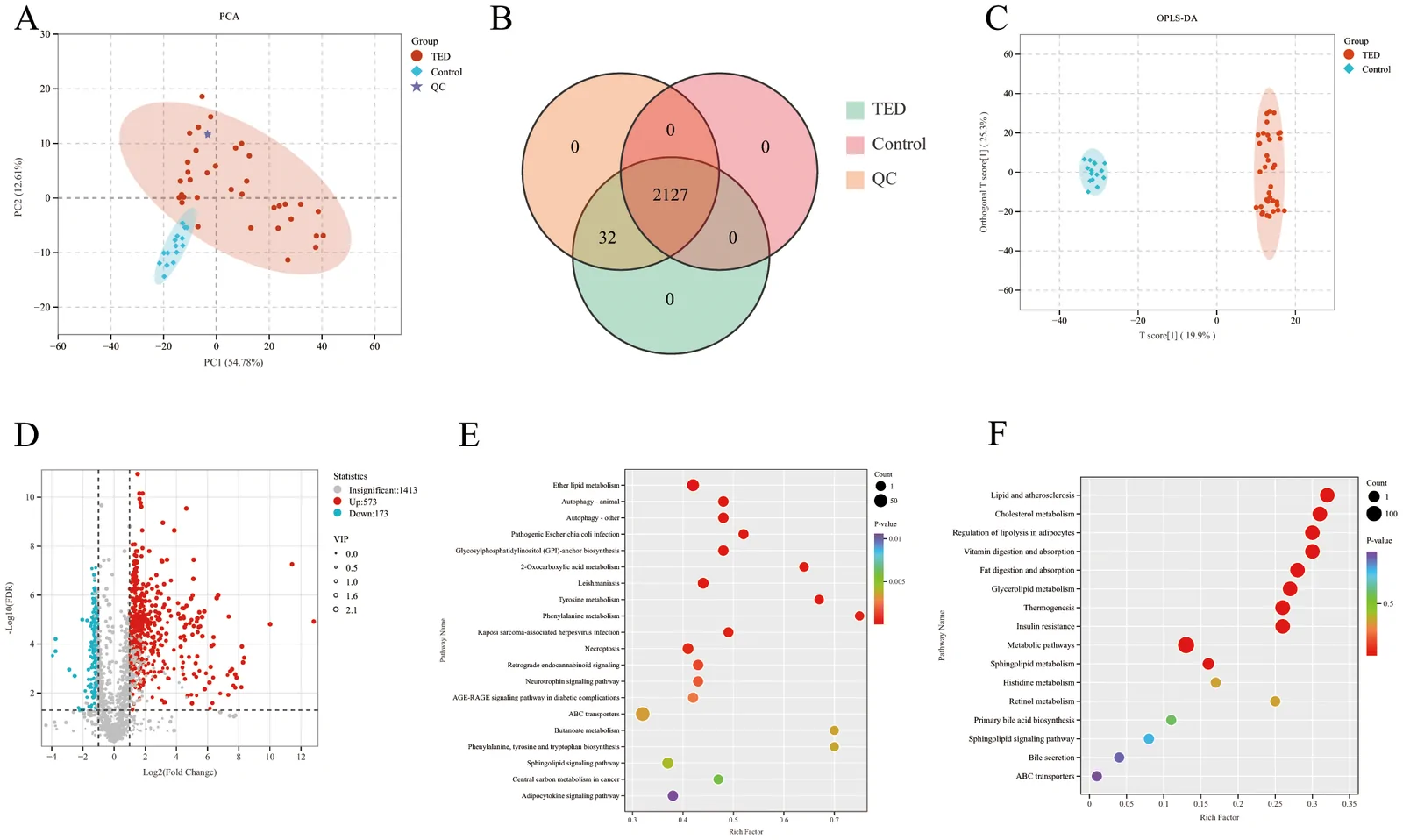
Illustrates the screening of differential metabolites and the subsequent metabolic pathway analysis. **(A)** The PCA plot displays the first principal component (PC1) on the horizontal axis and the second principal component (PC2) on the vertical axis. Each point corresponds to a sample, with distinct colors designating different groups: red for the TED group, blue for the control group, and purple for the QC samples. **(B)** This section presents the intersection of metabolites measured across the TED, QC, and control groups. **(C)** The OPLS-DA analysis model plot displays the x-axis as the predictive principal components and the y-axis as the orthogonal principal components, revealing a clear discrimination between the two groups. **(D)** The volcano plot utilizes the criteria of |log2FC| > 1, VIP score > 1, and P < 0.05. In this plot, blue dots signify downregulated metabolites, red dots denote upregulated metabolites. **(E, F)** These panels depict pathway analysis results based on the KEGG database.

PCA revealed significant differences in sample distribution patterns between the TED and control groups. Notably, the quality control (QC) samples exhibited a concentrated and uniform distribution, indicating the reliability of the metabolomic sequencing quality (**Figure 8A**). By intersecting the metabolites measured in the TED, QC, and control groups, we identified 2, 158 shared metabolites for subsequent analysis (**Figure 8B**). Next, orthogonal partial least squares-discriminant analysis (OPLS-DA) was employed to assess the clustering of the two groups. The score plot in **Figure 8C** clearly demonstrates the successful discrimination between control and TED samples. The metabolomic profiling conducted in this study identified these 2, 158 intersecting metabolites. The metabolite composition included: alcohols and amines (47), aldehydes, ketones, and esters (23), amino acids and their metabolites (330), benzene and substituted derivatives (90), bile acids (19), carbohydrates and their metabolites (71), coenzymes and vitamins (22), fatty acids (FA, 197), glycerolipids (GL, 409), glycerophospholipids (GP, 368), heterocyclic compounds (57), hormones and hormone-related compounds (23), nucleotides and their metabolites (91 and 150, respectively), peptides (PR, 1), secondary metabolites (SP, 238), steroids (ST, 5), tryptamines, cholines, pigments (5), and others (14). The volcano plot in **Figure 8D** indicates that, using the criteria of |log2FC| > 1, VIP score > 1, and false discovery rate (FDR)<0.05, 572 metabolites were upregulated and 173 metabolites were downregulated in the TED group.

To gain a deeper understanding of the metabolic dysregulation potentially underlying TED development, we utilized all metabolites with VIP scores > 1 to perform metabolite set enrichment and metabolic pathway analyses using the KEGG database. The metabolite set enrichment analysis revealed the top 20 significantly enriched metabolite sets (p < 0.05), including ether lipid metabolism, autophagy (animal and other), pathogenic Escherichia coli infection, glycosylphosphatidylinositol (GPI)-anchor biosynthesis, 2-oxocarboxylic acid metabolism and so on among the upregulated metabolites (**Figure 8E**). On the other hand, pathway analysis identified 16 metabolic pathways associated with downregulated metabolites. These pathways encompass lipid and atherosclerosis, cholesterol metabolism, regulation of lipolysis in adipocytes, vitamin digestion and absorption and so on (**Figure 8F**).

### Screening key differential metabolites

**Figure 9:**
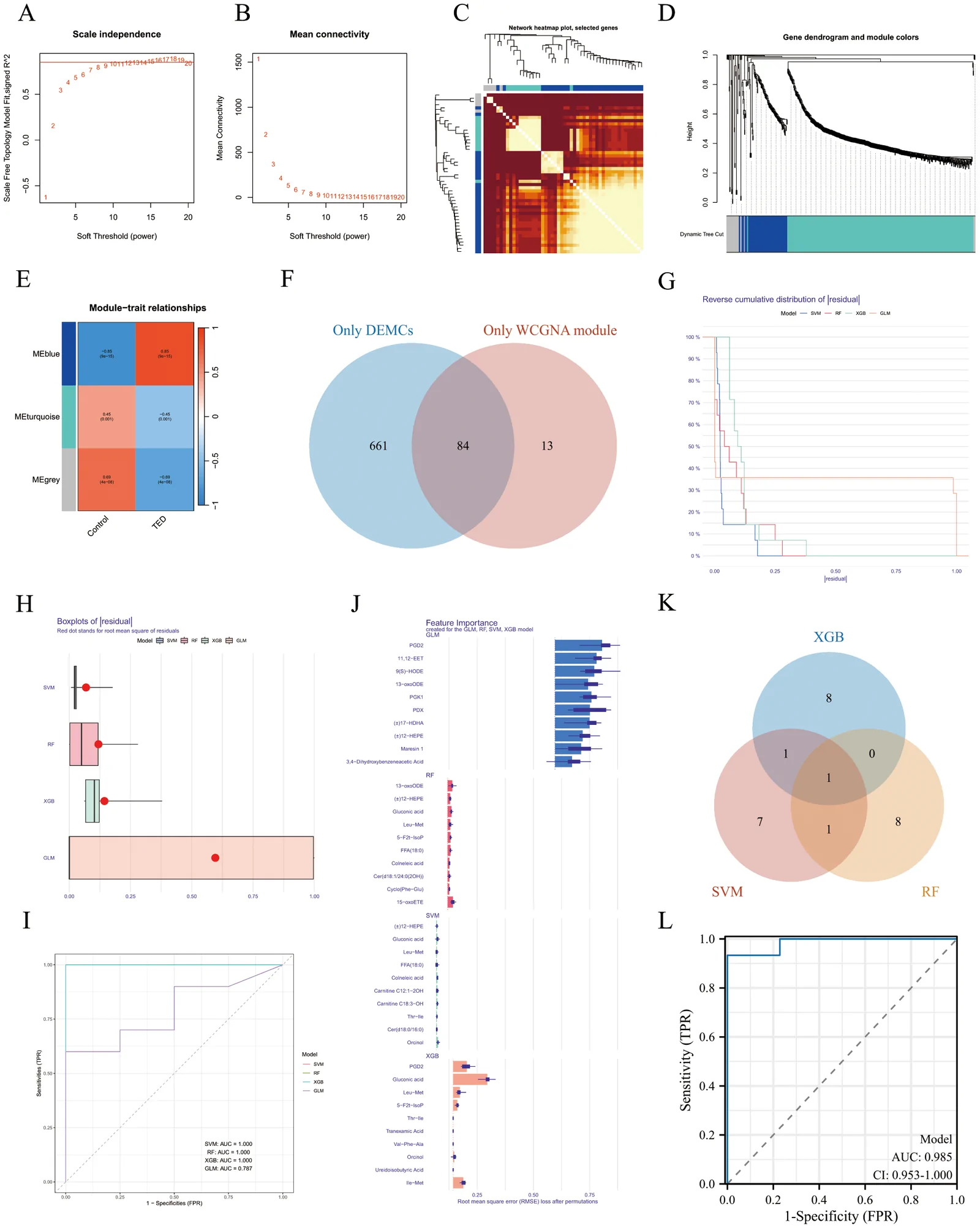
Screening and analysis of differential metabolites. **(A, B)** Optimization of network parameters via soft-threshold selection to ensure scale-free topology. **(C)** Heatmap depicting inter-module relationships and their connectivity patterns. **(D)** Hierarchical clustering dendrogram with dynamic tree-cutting for module identification. **(E)** Module-trait correlation matrix illustrating the strength of associations between modules and phenotypic traits. **(F)** Identification of overlapping metabolites by intersecting differentially expressed metabolites with those from the WGCNA blue module. **(G)** Comparative assessment of model performance using reverse cumulative distribution curves. **(H)** Distribution of residual errors across different machine learning algorithms. **(I)** ROC curve analysis evaluating the discriminatory capacity of RF, SVM, XGB, and GLM classifiers. **(J)** Ranking of the top 10 features by importance across the computational frameworks. **(K)** Determination of key metabolites through intersection of the top 10 metabolites identified by the RF, SVM, and XGB machine learning models. **(L)** Validation ROC curve demonstrating the predictive accuracy of the model within our study cohort.

We employed the WGCNA package to investigate module metabolites distinguishing the TED group from the control group. Co-expression modules were delineated by setting the soft power threshold at 10 and ensuring a scale-free topology fit index (R²) of 0.9 (**Figures 9A, B**). Subsequently, module merging was performed, and the dynamic tree cutting algorithm identified three distinct modules, each denoted by a unique color. The topological overlap matrix (TOM) was utilized to compute correlations, enabling the analysis of interactions among the three co-expression modules (**Figures 9C–E**). Correlation analysis revealed a significant association between the TED and control groups within the blue module, which encompassed 98 metabolites.

By intersecting the 746 differentially expressed metabolites (DEMs) with those from the WGCNA blue module, we identified 85 overlapping metabolites for subsequent machine learning validation (**Figure 9F**). Four machine learning frameworks—GLM, RF, XGB, and SVM—were constructed to evaluate their predictive metabolites. Notably, the GLM exhibited larger residuals and higher error rates compared to the other three models. Meanwhile, the GLM had a lower diagnostic AUC of only 0.787, and cross-validation directly comparing multiple model performances, along with calculated residual standard errors that exceeded those of the other models by more than twofold; thus, metabolites ranked in the top 10 by GLM were excluded from further analysis (**Figures 9G, H**). Receiver operating characteristic (ROC) analysis demonstrated varying discriminatory capacities among the models, with GLM achieving an AUC of 0.787, while SVM, RF, and XGB attained an AUC of nearly 1.0 (**Figure 9I**). We selected the top 10 metabolites from the SVM, RF, and XGB models (**Figure 9J**) and considered metabolites that appeared in at least two models as key biomarkers. Ultimately, gluconic acid, colneleic acid, and ile-Met were identified as critical metabolic substances implicated in the pathogenesis of TED (**Figure 9K**). Using the expression levels of these three metabolites, we constructed a predictive model that yielded an AUC of 0.985 (95% CI 0.953-1.0) in cohort validation (**Figure 9L**).

### Results of integrated transcriptomic and metabolomic analysis

**Figure 10:**
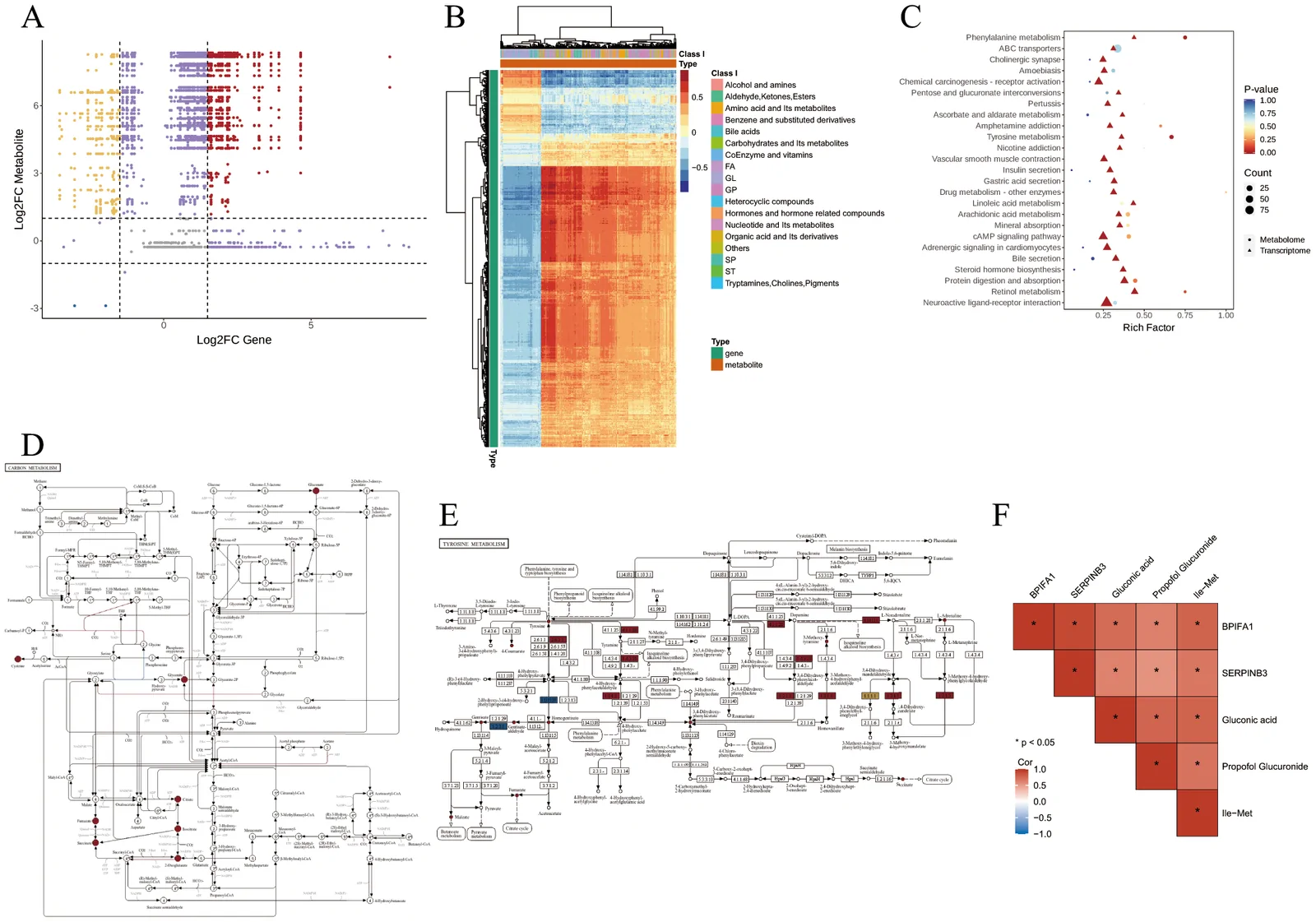
Integrated transcriptomic and metabolomic analysis visualization. **(A)** A nine-quadrant plot was employed to present the results of the combined metabolomic and transcriptomic analysis, offering a comprehensive overview of the interplay between gene expression and metabolite abundance. **(B)** The heatmap predominantly showcases the correlations between various metabolite types and genes, highlighting the intricate relationships within the biological system under investigation. **(C)** A bubble chart was constructed to depict the top 25 KEGG pathways significantly implicated by differentially expressed genes. In this chart, circles represent metabolites, while triangles denote genes. The color of each dot corresponds to the p-value, and the size reflects the quantity of differential genes or metabolites involved. **(D)** The carbon metabolism pathway. **(E)** The tyrosine metabolism pathway. **(F)** A heatmap is presented to demonstrate the correlations between two key genes and three key metabolites identified through machine learning screening, elucidating their potential regulatory roles and interactions.

Differential genes with |logFC| > 1.5 and metabolites with |logFC| > 1 were included in the analysis. A correlation coefficient threshold of > 0.8 was set, employing pearson correlation, and correlations with P < 0.05 were considered statistically significant. The integrated analysis incorporated transcriptomic and metabolomic data from 15 control samples and 35 TED samples. A nine-quadrant plot was utilized to display the outcomes of the integrated metabolomic and transcriptomic analysis. The x-axis represents the fold change in gene expression, while the y-axis indicates the fold change in metabolite abundance. Positive values denote an increase in metabolite abundance, whereas negative values signify a decrease. In the upper right quadrant, genes exhibit upregulation along with an increase in metabolite abundance. The lower right quadrant shows genes with upregulation but a decrease in metabolite abundance. The upper left quadrant contains genes with downregulation and an increase in metabolite abundance, while the lower left quadrant features genes with downregulation and a decrease in metabolite abundance. The central quadrant indicates non-significant changes in either genes or metabolites (**Figure 10A**). The heatmap primarily illustrates the correlations between different types of metabolites and genes, encompassing categories such as alcohol and amines, aldehyde, ketones, esters, amino acid and its metabolites, benzene and substituted derivatives, and bile acids (**Figure 10B**). Additionally, KEGG analysis was conducted, and the top 25 pathways based on enrichment scores were presented. Notably, the tyrosine metabolism pathway was significantly enriched, with a positive correlation observed between the changes in differential metabolites and genes, accompanied by a P-value < 0.05 (**Figure 10C**). Within the carbon metabolism pathway (**Figure 10D**), key differential metabolites identified through screening were enriched, and the expressions of OGDHL, ACSS1, HAO2, ENO4, and HK3 were affected (**Figure 10D**). Based on the enrichment ratio of pathways and their associations with diseases, the tyrosine metabolism signaling pathway emerged as the most crucial, with significant correlations between the enriched metabolites and genes (**Figure 10E**). Machine learning analysis revealed positive correlations between the expression of key genes BPIFA1 and SERPINB3 and key metabolites gluconic acid, colneleic acid, and ile-Met (**Figure 10F**).

### Verification of BPIFA1 and SERPINB3 expression levels

**Figure 11:**
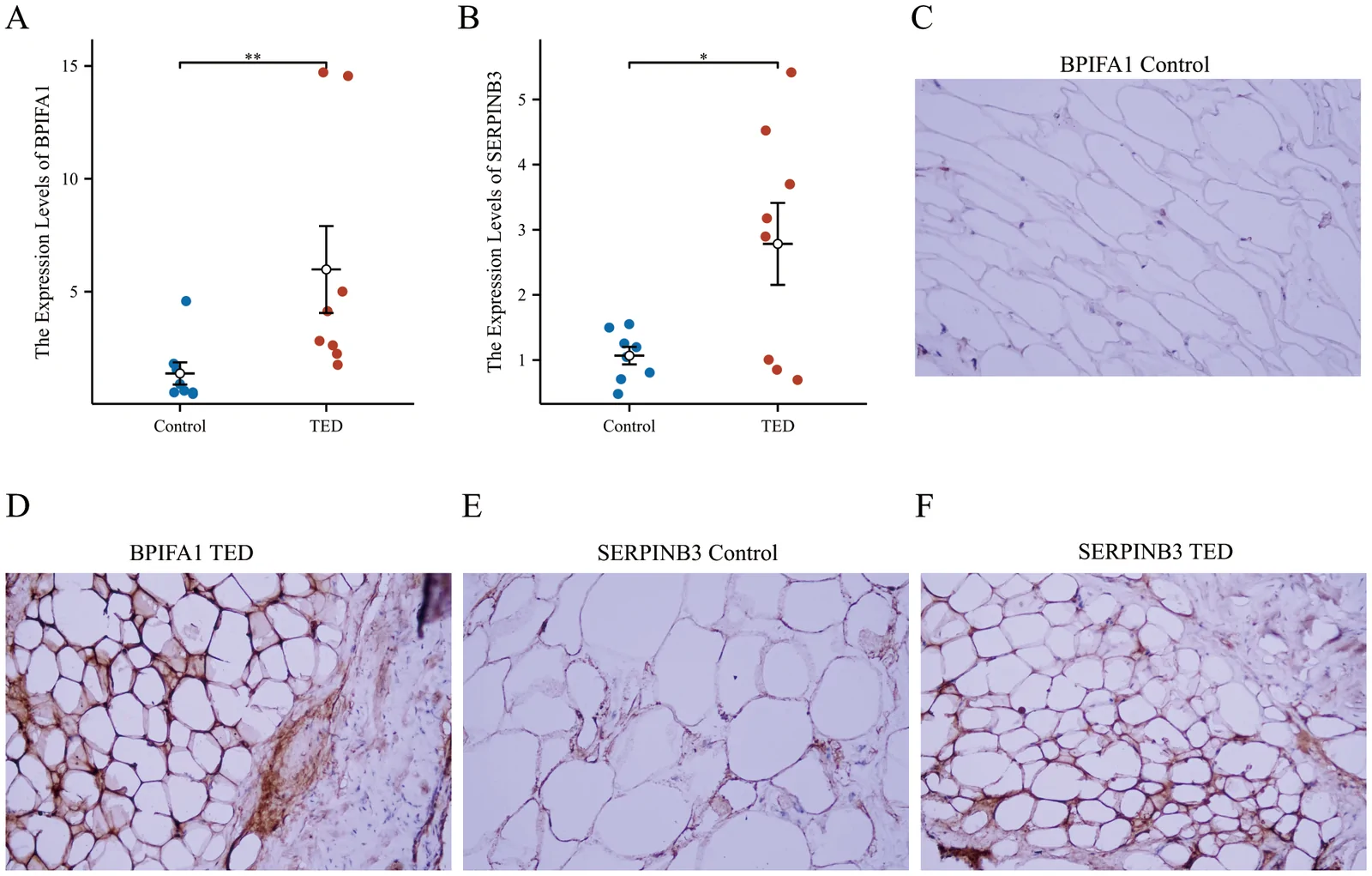
BPIFA1 and SERPINB3 expression levels. **(A, B)** The QPCR analysis demonstrates a statistically significant difference in the expression levels of the BPIFA1 and SERPINB3 gene between the orbital fat of patients with TED and those with orbital fat prolapse. **(C–F)** Immunohistochemical staining results for BPIFA1 and SERPINB3 indicate that the contents of these proteins are lower in the control group compared to the experimental group. This observation supports the hypothesis that the elevated expression levels of BPIFA1 and SERPINB3 may be linked to the pathogenesis of TED. *: P < 0.05; **: P < 0.01; ***: P < 0.001.

The QPCR analysis of the BPIFA1 gene revealed a statistically significant difference (p<0.05) in expression levels between the orbital fat of TED patients and that of patients with orbital fat prolapse (**Figure 11A**). This significant difference suggests that BPIFA1 may play a crucial role in the pathogenesis or pathological processes of TED. The expression level of SERPINB3 showed a statistically significant difference (p<0.05) in expression levels between the orbital fat of TED patients and that of patients with orbital fat prolapse (**Figure 11B**). The immunohistochemical results for BPIFA1 and SERPINB3 reveal that their contents in the control groups are lower than those in the experimental group (**Figures 11C-F**), we can observe that the expression levels of BPIFA1 and SERPINB3 are high in the adipose tissue around the orbit of the TED. This observation suggests that the elevated expression levels of these two genes may be associated with the pathogenesis of TED.

### Drug prediction and molecular docking

**Figure 12:**
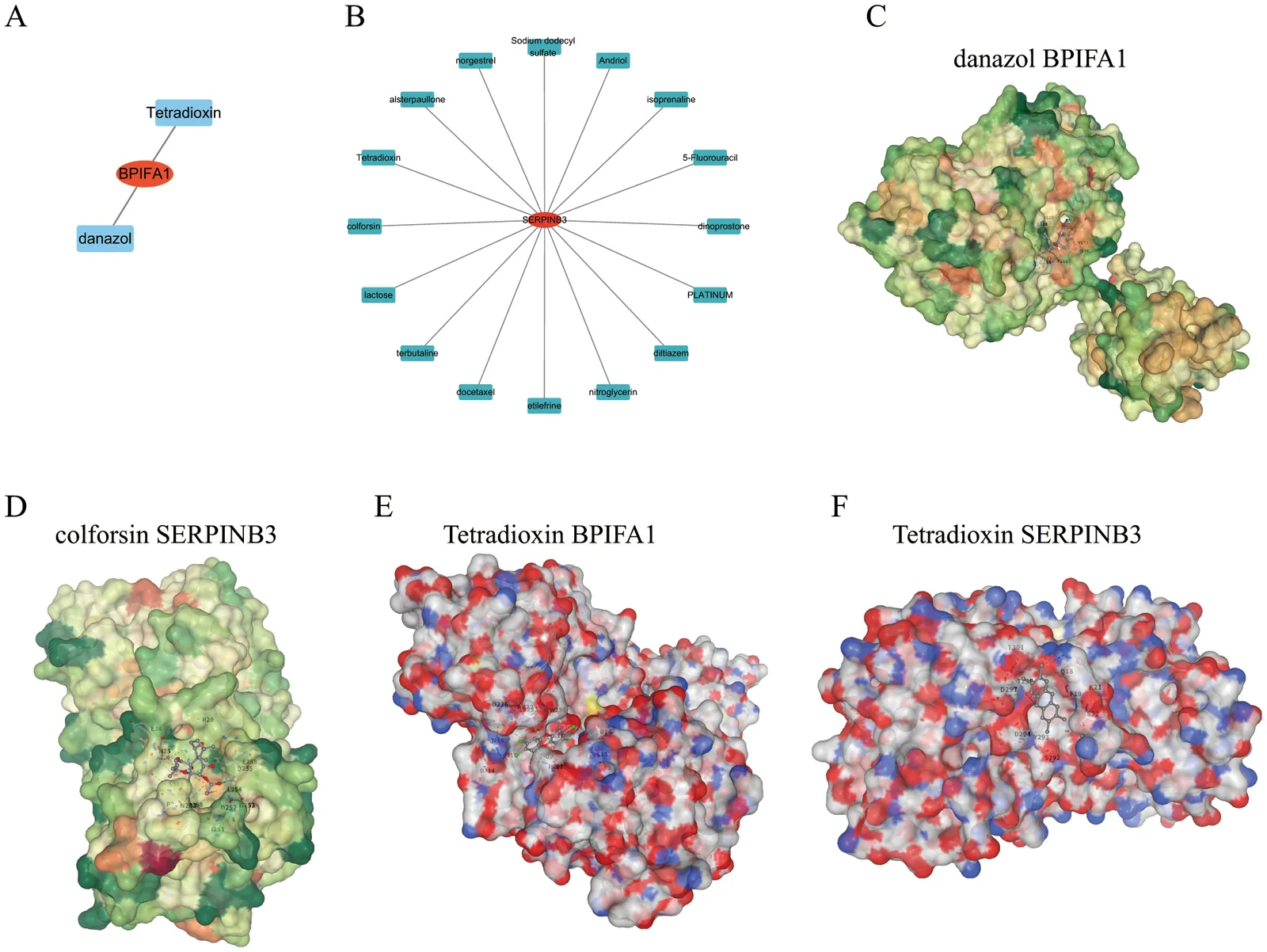
Interacting drugs candidates targeting feature genes in TED. **(A, B)** Comprehensive analysis was conducted using data from the DSigDB databases to identify interacting drugs candidates targeting the two feature genes, BPIFA1 and SERPINB3. **(C)** Danazol exhibited an even stronger affinity for BPIFA1, with a binding energy of -8.6 kcal/mol. **(D)** Colforsin was identified as a interacting drugs therapeutic agent for SERPINB3, showing a favorable binding energy of -7.4 kcal/mol. **(E, F)** Tetradioxin demonstrated the ability to interact with both SERPINB3 and BPIFA1, with binding energies of -6.3 kcal/mol and -6.7 kcal/mol, respectively.

To pinpoint interacting drugs that target the two identified feature genes, we conducted a comprehensive analysis using data sourced from the DSigDB databases. We undertook an extensive screening to identify the drug molecules, guided by P value < 0.05 as per the DSigDB database (**Figures 12A, B**). We conducted a rigorous screening process and identified BPIFA1 and SERPINB3 as crucial genes implicated in the pathogenesis of TED. Subsequently, we presented only the two drugs that significantly influenced BPIFA1 expression, including danazol and tetradioxin along with tetradioxin, which demonstrated the ability to jointly regulate both BPIFA1 genes. In the case of the SERPINB3 gene, our analysis revealed 16 drugs with statistical significance (P < 0.05), including tetradioxin. Among these, tetradioxin emerged as a prominent candidate, demonstrating interactions with all two feature genes and achieving a notable combined score of 108384.

Molecular docking simulations have been conducted to explore interactions for genes associated with TED. Danazol was identified as a potential binding drug for the BPIFA1 gene, exhibiting an even lower binding energy of -8.6 kcal/mol, suggesting a stronger affinity (**Figure 12C**). Specifically, colforsin emerged as an interacting drugs agent for SERPINB3, with molecular docking revealing a binding energy of -7.4 kcal/mol, indicating a favorable interaction (**Figure 12D**). Furthermore, tetradioxin demonstrated the ability to interact with both SERPINB3 and BPIFA1, with binding energies of -6.3 kcal/mol and -6.7 kcal/mol, respectively (**Figures 12E, F**). These findings underscore the potential of these compounds as targeted therapies for TED, with lower binding energies indicating a higher likelihood of effective molecular interactions.

### Results of MOFA multi-omics joint analysis

**Figure 13:**
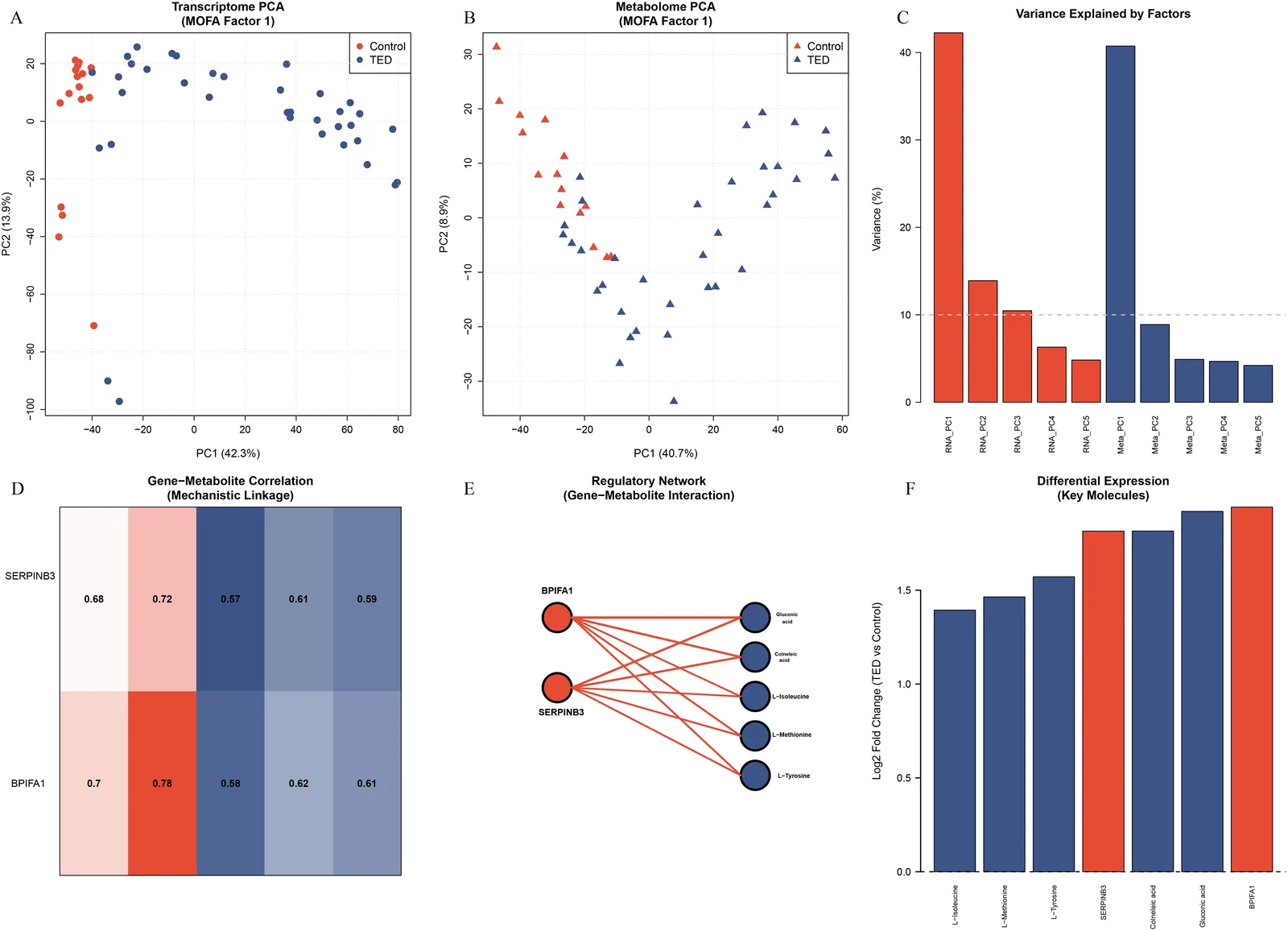
MOFA2 integrated analysis. Comprehensive PCA-based multi-omics integration overview. **(A)** Transcriptomic PCA showing the distribution of control (red) and TED (blue) groups in the PC1–PC2 space, with PC1 explaining 42.25% of the variance. **(B)** Metabolomic PCA, with PC1 explaining 40.74% of the variance; both omics layers display a consistent separation pattern. **(C)** Bar plot of explained variance, showing the contributions of the first five principal components. **(D)** Gene–metabolite correlation heatmap (including L-Tyrosine); all correlations are strong positive (*r* = 0.57–0.78), with increased margins to ensure complete label display. **(E)** Regulatory network, with genes as red nodes and metabolites as blue nodes; line colors indicate correlation direction, red represents the key genes of the transcriptome, while blue represents the key metabolites of the metabolome. **(F)** Differential expression bar plot showing the Log_2_FC of target molecules in TED, red columns represent the improvement factor of genes, and the blue ones represent the metabolic groups.

Transcriptomic principal component analysis revealed that PC1 explained 42.25% of the variance and PC2 explained 13.90%, while metabolomic PCA showed PC1 explaining 40.74% and PC2 explaining 8.89% of the variance, indicating a consistent disease-driven pattern across both omics layers (**Figures 13A–C**). The loadings of the target genes *BPIFA1* and *SERPINB3* on transcriptomic PC1 were 0.0352 and 0.0341, respectively, both with positive contributions, suggesting that they are key genes driving transcriptomic differences in TED and are significantly upregulated in the TED group. Pearson correlation analysis revealed that *BPIFA1* showed the strongest positive correlation with gluconic acid (*r* = 0.782, *p* = 2.05×10^-^¹¹), and was also significantly correlated with colneleic acid (*r* = 0.700, *p* = 1.57×10^-08^), L-tyrosine (*r* = 0.608, *p* = 2.77×10^-06^), L-methionine (*r* = 0.622, *p* = 1.42×10^-06^), and L-isoleucine (*r* = 0.581, *p* = 9.91×10^-06^). Similarly, *SERPINB3* exhibited strong positive correlations with all five metabolites (*r* range: 0.568–0.715, *p* < 5.24×10^-06^), and all correlations reached highly significant levels (*p* < 0.001) (**Figure 13D**). Tyrosine metabolism analysis identified 10 related metabolites, including L-tyrosine, DL-O-tyrosine, L-tyrosine methyl ester, and 3-iodo-L-tyrosine. Heatmap analysis showed a clear separation between control and TED samples (control samples clustered predominantly on the left, while TED samples clustered on the right), with most tyrosine metabolites exhibiting high expression in the TED group, indicating significant activation of the tyrosine metabolic pathway in TED (**Supplementary Figure 3A**). Boxplot analysis revealed that the five key metabolites (gluconic acid, colneleic acid, L-isoleucine, L-methionine, and L-tyrosine) and nine tyrosine metabolites were all significantly higher in the TED group than in the control group (*p* < 0.001, Wilcoxon rank-sum test), and all metabolites showed strong positive correlations with *BPIFA1*, *SERPINB3* expression (**Supplementary Figures 3B, C**). Collectively, these results demonstrate a core “two-gene–five-metabolite” statistically coupled multi-omics pattern centered on *BPIFA1*, *SERPINB3* in TED patients, where gene expression is tightly coupled with glucose metabolism (gluconic acid), fatty acid metabolism (colneleic acid), amino acid metabolism (L-isoleucine and L-methionine), and tyrosine metabolism (L-tyrosine), forming a stable inflammation–metabolic reprogramming axis (**Figure 13E**). The enrichment of this pathway suggests that tyrosine metabolism may be associated with the observed gene-metabolite correlation pattern; however, its biological mechanisms and pathological significance remain unclear and require further validation (**Figure 13F**).

## Discussion

TED also known as Graves’ ophthalmopathy, is the most common extrathyroidal manifestation of Graves’ disease, affecting a significant proportion of patients with hyperthyroidism. The pathogenesis of TED is characterized by an autoimmune inflammatory response targeting the orbital tissues, leading to symptoms such as proptosis, diplopia, and in severe cases, visual impairment. It is estimated that approximately 25% of patients with Graves’ disease will develop TED during their lifetime, with a notable impact on their quality of life due to the physical and psychological burdens associated with the disease (42). Although the exact mechanisms of TED remain inadequately understood, current research suggests a complex interplay between genetic predisposition, environmental triggers, and the dysregulation of the immune system (43). Traditional therapeutic approaches for the condition, encompassing corticosteroids, immunosuppressants, antibody drugs, and surgical procedures, frequently produce inconsistent outcomes and may fall short of effectively targeting the fundamental disease mechanisms. Even with the advent of newer antibody drugs, a subset of patients either exhibit non-responsiveness to the treatment or experience a relapse in therapeutic effects post-treatment. This underscores the urgent necessity for enhanced therapeutic strategies and a more profound comprehension of the molecular underpinnings of TED (5, 42).

Surgical operations can directly obtain tissue samples of the disease, and by using multi-omics sequencing methods, the underlying mechanisms of the disease can be further elucidated. In this study, we aim to explore the molecular mechanisms underlying TED by integrating transcriptomic and metabolomic analyses of orbital fat tissues from patients diagnosed with the disease. This multifaceted approach allows for a comprehensive understanding of the unique molecular alterations associated with TED, including dysregulated metabolic pathways and immune cell infiltration patterns. Previous studies have primarily focused on the immune and inflammatory aspects of TED; however, there is a notable gap in research regarding the systemic molecular characteristics and metabolic abnormalities present in the orbital fat tissues (13, 15). The analysis of gene expression in TED yielded significant insights regarding the underlying molecular mechanisms. A total of 4, 250 DEGs were identified, with notable upregulation of key genes such as BPIFA1 and SERPINB3. At the same time, we also used QPCR and immunohistochemistry to verify the expression of the gene, and found that the two genes were highly expressed in the TED group. These genes were confirmed as pivotal in the pathogenesis of TED, supported by WGCNA, Lefse and machine learning algorithms that yielded a diagnostic model with a high area under the curve (AUC) of 0.953 in internal validation and 0.717 in external validation using the GSE58331 dataset. BPIFA1 and SERPINB3’s roles as hub genes indicate their potential involvement in the regulatory networks that govern the inflammatory processes characteristic of TED. We calculated the Brier scores for the two datasets, with a Brier score of 0.154 for PRJNA1314138 and 0.2136 for GSE58331. In our dataset, adipose tissue from Chinese patients with orbital fat prolapse was used as controls, and adipose tissue from TED patients served as the experimental group. In contrast, the GSE58331 dataset comprised samples from a US population, with control tissues obtained from orbital puncture biopsies of normal individuals and experimental tissues from TED patients undergoing biopsy. The differences in diagnostic performance may be attributable to variations in population, disease severity, and sampling methods. The results deepen our understanding of the molecular pathology of TED, suggesting that these genes may serve as valuable biomarkers for early diagnosis and targeted therapeutic interventions, potentially facilitating the identification of patients at risk for severe disease progression (44, 45). BPIFA1 is primarily expressed in epithelial cells of the upper respiratory tract, lacrimal gland, and salivary gland, where it exerts antimicrobial functions and regulates ion transport (46). SERPINB3, a member of the serine protease inhibitor family, is expressed in various epithelial tissues, including skin, lung, and esophagus. Nevertheless, its elevated expression in whole tissue lysates indicates a significant inflammatory or epithelial remodeling component in TED. In TED, orbital fibroblasts may upregulate SERPINB3 upon activation, and immune cells such as macrophages and T cells can also express this gene to modulate protease activity (47). The functional enrichment analysis of the DEGs revealed their involvement in 694 biological processes, predominantly related to cellular structure and signaling pathways. Additionally, the significant pathways identified, including cytochrome P450 metabolism and steroid biosynthesis, suggest metabolic dysregulation as a critical aspect of TED progression. These findings not only elucidate the cellular mechanisms involved in TED but also point toward potential therapeutic targets, such as metabolic pathways that could be modulated to mitigate disease severity (48).

The examination of immune cell infiltration in TED has unveiled significant alterations in the local immune microenvironment. By employing the CIBERSORT analytical method, we observed an elevation in the populations of naïve B cells, plasma cells, and M1 macrophages within TED tissues, coupled with a concurrent decline in M2 macrophages. Given that M2 macrophages are typically associated with immunosuppressive functions, we hypothesize that in normal orbital fat, M2 macrophages predominate, playing a crucial role in maintaining immune homeostasis. However, during the onset of TED, the reduction in M2 macrophage levels and the corresponding increase in M1 macrophages likely amplify immune responses, thereby exacerbating orbital inflammation (49, 50). Furthermore, the correlation between the expression patterns of BPIFA1 and SERPINB3 and specific immune cell types implies that these genes may exert influence over immune reactions and inflammatory processes within the orbital milieu. This observation prompts intriguing inquiries into the functional contributions of these immune cell subsets to the pathogenesis of TED and their viability as potential therapeutic targets. Delving into the interplay between pivotal genes and immune cell dynamics holds promise for unveiling novel pathways for immunomodulatory therapies. Such therapies could be designed to re-establish equilibrium within the immune microenvironment of TED patients, thereby offering new hope for more effective treatment strategies (51).

The integration of metabolomic analysis further enhanced our understanding of TED’s pathophysiology. A total of 2, 158 metabolites were evaluated, with 572 showing significant upregulation and 173 downregulation. The identification of metabolic pathways, particularly those related to ether lipid metabolism and amino acid metabolism, indicates that metabolic dysregulation is intricately linked to TED pathogenesis. The predictive model based on key metabolites such as gluconic acid and phenylpropanolamine achieved an impressive AUC of 0.985, highlighting the diagnostic potential of metabolic profiling in TED. These findings underscore the importance of a multi-omics approach, combining transcriptomic and metabolomic data, to provide a comprehensive view of the disease mechanisms, ultimately paving the way for novel biomarkers that can aid in early detection and personalized treatment strategies for TED (52). In TED orbital tissues, mitochondrial dysfunction and accumulation of reactive oxygen species (ROS) are present, and gluconic acid may be involved in regulating redox balance. Moreover, gluconic acid may indirectly participate in orbital fibrosis by affecting fibroblast viability or inflammatory signaling pathways (53). The research on the relationship between colneleic acid, isoleucine methionine and diseases is still lacking in reports. The multi-omics analysis conducted in this study provides a rich framework for understanding the complex interplay of genetic, immune, and metabolic factors in TED. The identification of BPIFA1 and SERPINB3 as central genes, alongside the elucidation of significant immune cell changes and metabolic pathways, offers valuable insights into the disease’s pathophysiology. The current analysis cannot determine the directional relationship between genes and metabolites, it cannot distinguish whether genes regulate metabolites, metabolites regulate genes, or both are independently driven by common upstream factors (such as inflammation, hypoxia, or tissue remodeling). The directional relationships between BPIFA1, SERPINB3, and tyrosine metabolism-related metabolites need to be established through subsequent functional experiments, such as CRISPR-mediated knockdown or knockout of BPIFA1, SERPINB3 combined with metabolomics analysis, or metabolite treatment combined with gene expression assays. But the findings of this study are most directly applicable to inactive TED, and their generalizability is limited by disease activity, future validation in cohorts of active TED patients is required. Our multi-algorithm consensus strategy identified 2 hub genes, providing a robust transcriptomic signature that complements previous TED investigations. Unlike prior single-cell studies revealing GAS6-AXL-mediated fibrosis through PDGFRα+ DPP4+ fibroblast-M2 macrophage crosstalk, our bulk transcriptomic analysis captured broader gene expression changes across TED patients (47). Notably, partial hub genes (KRT19, SERPINB3, CFD) overlapped with fibrosis-related markers identified in previous single-cell studies, validating our consensus strategy. Compared to prior metabolomic and transcriptomic studies, we identified a novel candidate biomarker BPIFA1 not previously reported, expanding the TED diagnostic panel. While previous metabolomic studies highlighted proline and short-chain fatty acids, our metabolomic analysis provided complementary coordinated gene-metabolite signature insights, discovering novel metabolites including gluconic acid, colneleic acid, and isoleucine-methionine (13). Future studies integrating single-cell, metabolomic, and machine learning approaches would enhance biomarker discovery and therapeutic target identification in TED. Future research should focus on validating these findings through *in vivo* studies and exploring the therapeutic implications of targeting identified pathways, thereby advancing the clinical management of TED and improving patient outcomes (52, 54).

### Limitations

The present study, while providing valuable insights into the molecular mechanisms underlying TED, is not without its limitations. This study has several limitations. Orbital fat prolapses and autoimmune orbital diseases are fundamentally different in pathophysiology. However, owing to sampling constraints, the control group may also involve pathological features such as aging and orbital septum weakness. Future studies could adopt a more rigorous control group design, for example, using orbital tissues from patients with orbital cavernous hemangioma or non-inflammatory orbital diseases as controls to help distinguish the pathological processes. Orbital fat prolapse is more common in the elderly and may affect their status as a control group. One significant constraint is the absence of *in vitro* or *in vivo* experimental validation of the findings, which restricts the depth of mechanistic exploration. It is of utmost importance to note that our study has found associations between the expression levels of BPIFA1 and SERPINB3 and the patterns of immune infiltration in TED. In this study, patients with TED in a relatively stable state and a GAS score of less than 3 were selected for orbital decompression surgery. To detect the orbital tissues with a GAS score greater than 3, the obtained findings could only reveal the molecular characteristics of patients with TED in a stable state. Additionally, the research lacked comprehensive integration of clinical pathological features with molecular data, which could have provided a richer context for understanding the implications of the identified molecular signatures. We estimated the immune cell infiltration in orbital adipose tissue using CIBERSORT based on bulk RNA-seq data. However, we indeed lack *in vivo* experiments and single-cell sequencing to explain the relationship between the abundance of immune cells and hub genes. Future studies need to conduct experimental verification and single-cell sequencing to clarify this relationship. Although this study employed MOFA as the primary integration framework, other methods such as MOFA+ or SNF may complement the understanding of data structure from different perspectives. Future work could consider multiple integration frameworks for cross-validation and to broaden the scope of discovery. Finally, although efforts were made to standardize data across multiple datasets, the potential for batch effects remains a concern that could introduce bias in the results. Addressing these limitations in future investigations will be crucial for validating the findings of this study and enhancing the translational potential of the identified molecular markers and therapeutic candidates.

## Conclusions

In conclusion, this study employs a multi-omics approach, integrating transcriptomic and metabolomic analyses to elucidate the critical differential expression genes and metabolites associated with TED. The identification of BPIFA1 and SERPINB3 as hub genes, alongside gluconic acid, colneleic acid, and isoleucine-methionine as key metabolites, underscores the complex interplay between genetic and metabolic factors in TED pathogenesis. Additionally, immune cell infiltration analysis has shed light on the immune microenvironment’s role in the disease, highlighting the potential for novel therapeutic interventions targeting these pathways. Future research should focus on experimental validation and the integration of clinical datasets to propel these findings toward clinical application, ultimately aiding in the early diagnosis and effective management of TED.

## Data Availability

The datasets presented in this study can be found in online repositories. The names of the repository/repositories and accession number(s) can be found below: https://www.ncbi.nlm.nih.gov/, PRJNA1314138 https://www.ebi.ac.uk/metagenomics/, MTBLS15339.
